# Synthesis and evaluation of haemocompatible, antioxidant, and antibacterial properties, and acute oral toxicity of a methacrylic acid mediated glucoxylan for wound healing applications

**DOI:** 10.1371/journal.pone.0356647

**Published:** 2026-08-27

**Authors:** Samia Yaqoob, Arshad Ali, Fatima Akbar Sheikh, Muhammad Tahir Haseeb, Hassan Ali, Nasir Assad, Muhammad Farid-ul-Haq, Muhammad Imran, Muhammad Fakhar-e-Alam, Ahmed Vandy

**Affiliations:** 1 Faculty of Sciences, Superior University Lahore, Lahore, Pakistan; 2 Institute of Chemistry, University of Sargodha, Sargodha, Pakistan; 3 College of Pharmacy, Niazi Medical and Dental College, Sargodha, Pakistan; 4 College of Pharmacy, University of Sargodha, Sargodha, Pakistan; 5 Department of Physics, University of Agriculture Faisalabad, Faisalabad, Pakistan; 6 International Center for Interdisciplinary Research in Sciences, The University of Lahore, Lahore, Pakistan; 7 Chemistry Department, Faculty of Science, King Khalid University, Abha, Saudi Arabia; 8 Department of Physics, Government College University Faisalabad, Faisalabad, Pakistan; 9 Faculty of Pharmaceutical Sciences, College of Medicine and Allied Health Sciences, University of Sierra Leone, Freetown, Sierra Leone; Al-Azhar University, EGYPT

## Abstract

Herein, a copolymeric hydrogel comprising a naturally occurring swellable biopolymer, *i.e.*, glucoxylan (a major polysaccharide of chia seed mucilage (CM)) and methacrylic acid (MA), was synthesized through free radical polymerization for wound healing applications. The formation of CM-co-MA was authenticated through FTIR spectroscopy. The CM-co-MA appeared non-hemolytic and non-thrombogenic, and exhibited noteworthy antioxidant potential with DPPH scavenging activity ranging from 18.68 to 47.70 µg AAE µg/mL, FRAP activity from 32.22 to 63.70 µg AAE µg/mL, TFC from 27.91 to 73.59 µg QE/mL, and TPC from 26.95 to 58.21 µg GAE/mL. The CM-co-MA showed antibacterial activities against *Listeria monocytogenes* (Gram-positive) and *Escherichia coli* (Gram-negative), having a zone of inhibition (ZOI) of 27.87 and 23.00 mm, respectively. The antifungal activities were recorded against *Aspergillus flavus* and *Candida albicans,* and found the length of growth inhibition of 72 and 76 mm, respectively. The acute oral toxicity study, acute dermal toxicity, and eye irritation test demonstrated the non-toxic, innocuous, and non-irritant attributes of the CM-co-MA, respectively. All animals stayed alive without any significant abnormalities during the 14 day study, and all hematological and biochemical parameters of the control and experimental group animals were comparable. Histopathology of the vital organs of rabbits also showed normal cellular architecture without any abnormalities. A wound healing study on rabbits revealed the tissue regeneration, with 84.40% recovery in treated rabbits after 14 days, and comparable with the positive control. Hence, the CM-co-MA is considered a non-toxic material having haemocompatibility, antioxidant, antibacterial, and antifungal activities for potential application in wound healing.

## Introduction

The skin is the largest organ in the human body, which makes up around 15% of the total body weight. It serves as a barrier to protect the body from bacterial infections and environmental. The ordinarily resilient barrier of the skin is often compromised by various types of trauma, burns, ulcers, skin infections, injuries and other detrimental events, which also disrupt the vital function of the skin in sensory perception [1–3]. Therefore, skin care and wound healing are among the most critical topics in the health care system. Wound management has been transformed from conventional to nanodevices and from passive to smart dressing [4–6].

A wound is defined as any disruption of the biochemical, cellular, or structural integrity of the skin or underlying tissues. Hemostasis, inflammation, proliferation, and maturation (remodeling) are the four concurrent stages of wound healing, which is a complicated biological and molecular process [7]. Hydrogel-based wound dressings stand out among other types of dressings because of their capacity to speed up the healing process [8]. They keep the wound site moist and act as a barrier against microbes, encouraging effective tissue recovery. One reliable technique for producing hydrogels is to introduce crosslinkers into the polymer structure, which enhances not only the mechanical strength of the hydrogel but also induces important physicochemical properties for wound management [9].

The polysaccharide based hydrogels of natural origin are three-dimensional network systems with hydrophilic functional groups that retain a large amount of water [10]. They are biocompatible in nature and possess antibacterial, antitumor, immune, and antioxidant activities [11–14]. They have been extensively utilized in tissue engineering, [3] for the development of nanofibers, [15] wound healing, development of novel drug delivery systems, and various biomedical applications [16–19]. The resistance of hydrogels to water dissolution is a result of the cross-linking of their polymer chains. Hydrogels can be prepared using natural, synthetic, and semisynthetic polymers. Among these, naturally occurring polymers, especially swellable polysaccharide-based biopolymers, have gained much attention from researchers owing to their porous structure, non-toxic nature, biocompatibility, biodegradability, low nonimmunogenicity, stimuli-responsive properties, chemically modifiable nature, and resemblance to the natural extracellular matrix [20–24].

Chia (*Salvia hispanica*) seeds have substantial nutritional and medicinal significance owing to their diverse constituents [25] Chia seeds are rich in proteins, fats, carbohydrates, minerals, and dietary fibers [26,27]. Upon soaking in water, chia seeds release a thick mucilage (CM) covering the outer surface of the seeds that is mainly composed of glucoxylan as a major polysaccharide [28,29]. Previously, CM has been reported as a stimulus-responsive and sustained drug release matrix with and without chemical modification [30,31]. However, the novelty of this study lies in the combination of an optimized hydrogel design and extensive preclinical biological evaluation. This study aim to synthesizes a coplymeric hydrogel of chia seed mucilage with methacrylic acid (CM-co-MA) and systematically evaluates its hemocompatibility, antioxidant activity, antimicrobial efficacy, toxicity, and *in vivo* wound healing potential, which have not been reported in prior studies. This integrated approach provides a comprehensive understanding of the therapeutic potential of the hydrogels and addresses key preclinical requirements for wound healing applications.

## Materials and methods

### Materials

Chia seeds were purchased from a local market in District Sargodha. *N-N*-methylenebisacrylamide (MBA, ≥ 99%, Sigma-Aldrich, Germany), methacrylic acid (MA, 99%, Sigma-Aldrich, Germany), ammonium persulfate (APS, ≥ 98%, Sigma-Aldrich, Germany), *n*-hexane (≥95%, Riedel-de Haen, Germany), and ethanol (≥98%, Riedel-de Haen, Germany) were used in this research work. All chemicals and reagents were of analytical grade and used without further purification. Distilled water (DW) was used to prepare the solutions/dispersions.

### Anesthesia and ethical compliance

In this study, sodium pentobarbital was used as the anesthetic agent. For blood collection, the rabbits were anesthetized with a dose of 30 mg/kg intraperitoneally (i.p.), sufficient to achieve surgical anesthesia without causing mortality. Euthanasia was performed at a higher dose of 60 mg/kg i.p. to ensure humane death. All experimental procedures adhered to the ARRIVE guidelines, ensuring responsible animal research. The study protocol was approved by the Institutional Research Ethics Committee of the Superior University Sargodha Campus, Pakistan (Ref: SU/SGD/IREC/25/0005), and all methods were performed in accordance with relevant guidelines and regulations.

### Isolation of CM

An aqueous extraction method was employed to extract the mucilage from chia seeds as reported in the literature [30]. Briefly, the cleaned and overnight-soaked chia seeds were heated for 1 h at 60°C. The extruded mucilage from chia seeds was separated using a nylon mesh and thoroughly washed three times with *n*-hexane and DW to purify it from lipophilic and hydrophilic impurities, respectively. The purified mucilage was then dried at 60°C, ground using a pestle and mortar, sieved through a 60-mesh sieve, and stored in an airtight container.

### Formulation of CM-co-MA

**Scheme 1:**
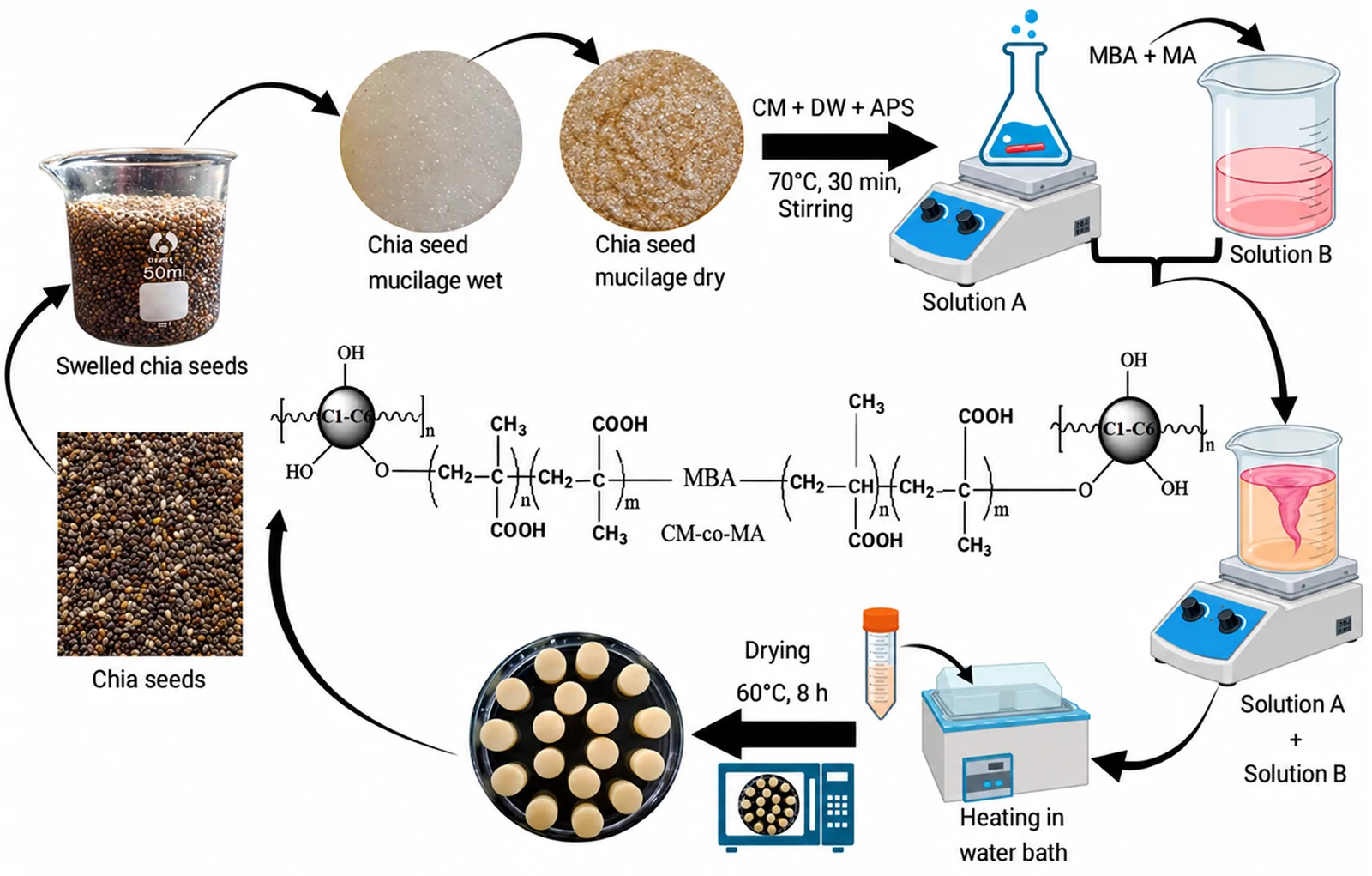
An illustration for the isolation of CM and the synthesis of CM-co-MA.

The CM was used to synthesize copolymer hydrogel, *i.e*., CM-co-MA, using MA as a monomer according to the procedure reported by Awais et al.[32]. The first step in the synthesis of CM-co-MA was the preparation of a CM aqueous suspension (2%, w/w, 100 mL). The APS (0.5% mole ratio of monomer, 100 mL) was added as the initiator in the CM aqueous suspension and named as “solution A”. In a separate flask, another solution (solution B) was prepared by adding MBA (0.5% mole ratio of monomer, 100 mL) to MA (30% w/w, 100 mL). The two solutions were mixed at room temperature and agitated for 30 min. The final mixture was poured into the test tubes and heated in a water bath for 8 h to obtain a transparent hydrogel. The formed hydrogel was separated from the test tube and cut into 5 mm thick discs. These discs were washed with an aqueous ethanol solution (30%, v/v) to remove the unreacted reagents and dried at 50°C in an oven for further experimental work. The procedure for isolating CM and synthesizing CM-co-MA is illustrated in Scheme 1.

### Characterization of CM-co-MA

FTIR spectroscopic analysis of CM and CM-co-MA was conducted to monitor the structural changes in CM after polymerization. The KBr disc method was used to record the FTIR spectra of both materials. Both CM and CM-co-MA were first ground to make a powder and then mixed separately with KBr. The mixture of each sample was compressed under hydraulic pressure to obtain thin discs of both materials, dried at 50°C for 30 min, and finally scanned using an IR Prestige-21 spectrophotometer (Shimadzu, Japan) in the range of 4000−400 cm^-1^ to record FTIR spectra.

### Haemocompatibility studies

The hemolytic index and thrombosis were determined to assess the hemocompatibility of CM-co-MA for potential biomedical applications. The procedure reported by the International Organization for Standardization (ISO) was followed to determine thrombosis and hemolytic potential.

### Thrombosis

The gravimetric method was used to determine the thrombogenicity of CM*-*co-MA [22,33]. A suspension of CM-co-MA was prepared by mixing a small quantity (500 mg) of the finely divided hydrogel in phosphate buffer saline (PBS). The resulting suspension was incubated at 37°C for 24 h. After incubation, the supernatant layer of PBS was decanted, and 0.2 mL of 0.1 M CaCl_2_ and 2 mL of citrate blood were added. A lapse period of 0.75 h was given to the mixture to prevent blood clotting. The DW (5 mL) and 37% of formaldehyde were added to fix the formed clots, followed by the separation of the formed clots, drying, and weighing the dried clots. The same procedure was performed without CM for the positive controls. For the negative control, the procedure was performed without CM or citrate blood. The concentration of thrombus formation (%) was calculated using Eq. 1.


$$\begin{array}{c} Thrombose\;(\%)=\frac{\text{Mass of test sample }-\text{ mass of }(-)\text{ control}}{\text{Mass of }(+)\text{ control }-\text{ mass of }(-)\text{ control}}\times \text{100} \end{array}$$ (1)

### Hemolytic potential

Direct or indirect contact of CM*-*co-MA with blood may occur when a CM-co-MA-based formulation is administered to humans. The compatibility of the CM-co-MA with the blood was determined by evaluating its hemolytic potential, as described by the American Society for Testing and Materials (ASTM) [34]. A 500 mg of CM*-*co*-*MA was placed in PBS and incubated at 37°C for 24 h, followed by washing with PBS. After subsequently, 2 mL of citrate blood was added to the mixture and placed in an incubator for 3 h at 37°C. The supernatant was separated after centrifugation for 15 min at 10^4^ rpm and then scanned using monochromatic radiation at 540 nm using a UV-Vis spectrophotometer to determine the optical density (OD). For the positive and negative controls, the same procedure was performed by incubating citrate blood with DW and PBS, respectively. Eq. 2 was used to evaluate the hemolytic potential of CM-co-MA.


$$\begin{array}{c} \text{Hemolytic index}=\frac{OD\;of\;test\;sample\;-\;OD\;of\;(-)\;control}{OD\;of\;(+)\;control\;-\;OD\;of\;(-)\;control}\times \text{1}\text{0}\text{0} \end{array}$$ (2)

### Determination of antioxidant activities

#### DPPH assay.

The DPPH radical scavenging assay was conducted following a previously reported procedure with slight modifications [35]. To the CM-co-MA suspension (100–500 µg/mL), 1 mL of 0.1 mM methanolic DPPH solution and 2 mL of methanol were added. The mixture was thoroughly mixed and allowed to settle at room temperature for 30 min in the dark. The absorbance was measured at 517 nm using a Shimadzu UV-1800 spectrophotometer. Ascorbic acid was used as the standard. Eq. 3 was used to determine the DPPH activities of both CM-co-MA and ascorbic acid.


$$\begin{array}{c} \text{DPPH }=\;\frac{\left(\text{Absorbance of control }-\text{ Absorbance of sample}\right)}{\text{Absorbance of control}}\times \text{100} \end{array}$$ (3)

#### FRAP assay.

The FRAP assay was performed with minor modifications to the method described by Assad et al. [36]. Briefly, 2.5 mL of sodium phosphate buffer (0.2 M, pH 6.6) and 2.5 mL of 1% potassium ferricyanide solution were mixed with 100–500 μg/mL of CM-co-MA suspension. Subsequently, the mixture was placed at 50°C for 20 min. Subsequently, a 10% w/v solution of trichloroacetic acid was added, and the mixture was centrifuged at 3000 rpm for 10 min. 0.1% ferric chloride solution and 2.5 mL of DW were added to the obtained supernatant. Absorbance was measured at 700 nm using a Shimadzu UV-1800 spectrophotometer. The FRAP potentials of CM-co-MA and the standard, *i.e*., ascorbic acid (500 µg/mL), were calculated using the standard curve method.

#### TPC determination.

The TPC was determined using the Folin–Ciocalteu method [37]. Briefly, 100–500 μg/mL of CM-co-MA were prepared in DW, and 250 μL of 1 N Folin-Ciocalteu reagent and 2 mL of DW were added to it. For 8 min, the mixture was placed at room temperature in the dark. Then 950 μL of DW and 750 μL of a 20% sodium carbonate solution were added. The resultant solutions were incubated in the dark for 30 min. Absorbance was measured at 765 nm using a Shimadzu UV-1800 spectrophotometer. Gallic acid was used as the reference, and the results were reported as mg of gallic acid equivalents (µg GAE) per mL of CM-co-MA, and TPC was determined using the standard curve method.

### TFC determination

Using a colorimetric method based on aluminum chloride (AlCl_3_), the proportion of flavonoids was ascertained in accordance with the protocol reported in the literature, with slight modifications [38]. Different concentrations, *i.e*., 100–500 μg/mL of CM-co-MA were combined with 0.75 mL of methanol, and the resultant volume was adjusted to 2 mL using DW. Sodium nitrite (5%, 300 μL) and AlCl_3_ (10%, 300 μL) were added to each sample, and the solutions were incubated for 10 min. After a specific time, NaOH (1 M, 2 mL) was added, and the final volume was adjusted to 5 mL using DW. The solutions were incubated at room temperature for 40 min. The absorbance was measured at 510 nm using a Shimadzu UV-1800 spectrophotometer. TFC was expressed as μg of quercetin equivalent per gram (μg QE/g) of CM-co-MA, with quercetin as the reference standard, and calculated using the standard curve method.

### Antibacterial activities

The antibacterial activities of CM-co-MA were evaluated against *Listeria monocytogenes* (gram-positive) and *Escherichia coli* (gram-negative) strains, both obtained from the American Type Culture Collection (ATCC). The test strains were cultured on Mueller–Hinton agar plates (Thermo Fisher Scientific, Waltham, MA, USA). For the assay, the bacterial culture medium was autoclaved, poured into Petri dishes, and incubated at 37°C for 24 h. The turbidity of the bacterial culture was adjusted, and antibacterial activity was assessed using the disc-well method described by Roheen et al [39]. Briefly, the culture medium was evenly spread on petri dishes, and 6 mm wells were prepared in the agar. CM-co-MA (40 µg/mL) was loaded into the first well (sample, labeled as “a”), ciprofloxacin (40 µg/mL) served as the positive control in the second well (labeled as “b”), and DW was used as the negative control in the third well (labeled as “c”). The plates were incubated at 37°C for 24 h, after which the ZOIs were measured. The overall procedure was performed in a laminar flow hood under aseptic conditions to ensure a clean environment for the experiment. The experiment was repeated three times, and the results are presented as the mean ± standard deviation (SD). CM-co-MA was tested at a single concentration (40 µg/mL) for antibacterial activity to provide an initial assessment of its bactericidal potential, consistent with previous studies on polysaccharide-based copolymers. Therefore, dose-response studies should be considered in future studies. Antioxidant assays, however were conducted over multiple concentrations (100–500 µg/mL) to allow quantitative comparison with standards and observe dose-dependent activity.

### Antifungal activities

The Antifungal activity of CM-co-MA was evaluated against *Aspergillus flavus* and *Candida albicans* using a test tube macro-dilution method, slightly adapted from the method described by Assad et al [40]. Spores from 3–5-day-old cultures grown on Sabouraud Dextrose Agar were harvested to prepare fresh fungal suspensions. Sterilized test tubes containing 5 mL of Sabouraud Dextrose Broth were inoculated with 1 mL of a standardized suspension (~1 × 10^6^ spores/mL). Three treatments were tested: (a) CM-co-MA (sample), (b) voriconazole (positive control), and (c) CM (negative control). After treatment, the final volume in each tube was adjusted to 6 mL using sterile DW. Tubes were incubated in a slanted position at 28 ± 2°C for 48 h to maximize surface area exposure and aeration. Following incubation, fungal growth in the treated and control tubes was visually assessed. A reduction or absence of visible fungal biomass was considered indicative of strong antifungal activity. The results are expressed as the mean length of growth inhibition along the tube ± SD, based on three independent replicates.

### Acute oral and dermal toxicity studies

#### Selection of animals.

The acute oral and acute dermal toxicity testing of CM-co-MA was carried out in accordance with the directives of the Organization for Economic Co-operation and Development (OECD) 420 and 402, respectively, on Swiss albino rabbits [41,42]. The animals were acquired from the animal house of the University of Sargodha and kept in hygienic cages, maintaining the temperature at 25°C, humidity of approximately 40%, and a photoperiod of 12 h. The entire procedure was performed following the guidelines of Good Laboratory Practices (GLP). The study protocol was approved by the Institutional Research Ethics Committee, Superior University Sargodha Campus, Pakistan (Ref: SU/SGD/IREC/25/0005, dated April 25, 2025). The animals were divided into four equal groups (n = 6) : control (CM1) and experimental (CM2, CM3, and CM4). All procedures for animal handling, anesthesia for blood collection (30 mg/kg, i.p.), and euthanasia (60 mg/kg, i.p.) were performed in compliance with institutional ethical guidelines. Blood samples were collected under anesthesia to minimize stress, and euthanasia was performed using the approved higher dose.

### Dosing to animals

Overnight-fasted animals in the experimental groups (CM2, CM3, and CM4) were administered three different doses of CM-co-MA, i.e.,: 0.05, 0.3, and 2 g/kg body weight, respectively. The control group (CM1) animals were not provided with any amount of CM-co-MA. All animals were deprived of food for at least 6 h after the administration of CM-co-MA but were provided water *ad libitum*. After 6 h, all the animals were maintained on a standard laboratory diet for 14 days.

### Behavioral observations

During the 14 days of acute oral toxicity studies, the animals were closely monitored for any symptoms of diarrhea, constipation, allergic reactions, fluctuations in respiration rate, drowsiness, irritation, lacrimation, mortality, behavioral changes, or any other unusual responses or conditions.

### Determination of body weight

The weights of the animals were calculated before and after CM-co-MA administration. The weights of each animal in the control and experimental groups were recorded for the next 14 days.

### Consumption of water

The water in graduated bottles was placed in the cages, and the consumption of water by the animals in all groups was recorded for 14 days.

### Intake of food

The animal diet was weighed and placed in animal cages in the morning. On the following morning, the unconsumed diet was removed from the cage and weighed again to calculate the weight of the consumed diet. These procedures were performed daily, and food intake was documented for 14 days.

### Hematology and biochemical analysis

On the 15^th^ day of the study, blood samples were collected from the control and experimental group animals after anaesthetization. Blood samples were withdrawn from the jugular artery of the rabbits. The collected blood samples were transferred into two tubes, one of which was lined with ethylenediamine tetraacetic acid. Complete blood count (CBC), including red blood cells (RBCs), white blood cells (WBCs), platelet count, mean corpuscular volume (MCV), mean corpuscular hemoglobin (MCH), will be determined. Blood serum analysis was conducted to measure urea, uric acid, cholesterol, creatinine, and liver function.

### Absolute organ weight and gross necropsy

Animals in all groups were sacrificed after successful collection of blood samples on day 15, and the weight and macroscopic analysis of their major and vital organs, such as the stomach, liver, small intestine, lung, heart, and kidney, were carried out to note the absolute organ weight and proceed to the histopathology evaluation, respectively.

### Histopathology evaluation

The tissues of the vital organs were cut into thin slices, stained with hematoxylin and eosin, and observed under a microscope to examine their cellular architecture.

### Acute dermal toxicity

The potential dermal toxicity of CM-co-MA was tested in six white albino rabbits. A thick aqueous slurry (200 mg/5 mL) of CM-co-MA was applied to the shaved hindlimb skin of the rabbits. Cotton gauze was placed on the applied slurry to avoid displacement. The gauze was removed every 24 h for the next 96 h and checked for any symptoms of redness, allergic reactions, infection, or any other abnormality.

### Primary eye irritation

Six rabbits were selected, and an aqueous dispersion of CM*-co-MA* (1 mg/3 mL) was prepared. A small volume was administered to the right eye of each rabbit, while the left eye was left untreated and served as a control. For the next 72 h, any signs of redness, lacrimation, inflammation, erythema, edema, lesions, or other abnormalities in the eyes were observed [43]. The Draize scale was used to determine the primary eye irritation score.

### Wound healing study

The animal house of the University of Sargodha provided 18 rabbits with their weight ranging from 1.5-1.7 kg, and they were divided into three groups (n = 6), *i.e*., control, standard, and sample. The studies of wound healing were conducted following the protocols laid out by the Institutional Research Ethics Committee, Superior University Sargodha Campus, Pakistan vide Ref: SU/SGD/IREC/25/0005, dated: April 25, 2025, under the National Institute of Health Guidelines for the Care and Use of Laboratory Animals (NIH Publiction No. 8023, revised 1978) and OECD guidelines (OECD, 2008). Before performing experimentation, animals were handled and caged according to the standard protocols as mentioned before. Afterward, all 18 rabbits were carefully anesthetized, and the hair of their hind limbs was removed. Standardized wounds were surgically induced on the shaved hind limbs of each rabbit. The animals of the control group were left without any treatment or bandage. The wound of the standard group animals was covered with marketed bandage (*Sufre tulle*^®^), and those of the sample group animals were covered with CM-co-MA bandage. For observation of the healing process, a raw wound area was sketched on the paper. The healing process was monitored daily until complete healing.

Excised wound tissues from all experimental groups were fixed in 10% formalin, embedded in paraffin, sectioned at 5 µm thickness, and stained with hematoxylin and eosin (H&E). The sections were examined under a light microscope to evaluate re-epithelialization, collagen deposition, dermal organization, and inflammatory cell infiltration. Representative images were captured for the negative control, CM-co-MA-treated, and positive control groups on day 14. These histological observations complemented the quantitative wound closure assessments.

### Statistical analysis

The mean values of all parameters were recorded and presented along with the SD. Statistical significance was evaluated using One-Way Analysis of Variance (ANOVA) followed by Tukey’s Honestly Significant Difference (HSD) test for all-pairwise comparisons. All data were analyzed using Statistix 8.1 software. A significance level of p<0.05 was applied to determine statistical differences, where different superscript letters indicate significantly different means.

## Results and discussion

### Synthesis and FTIR spectroscopic analysis

**Fig 1:**
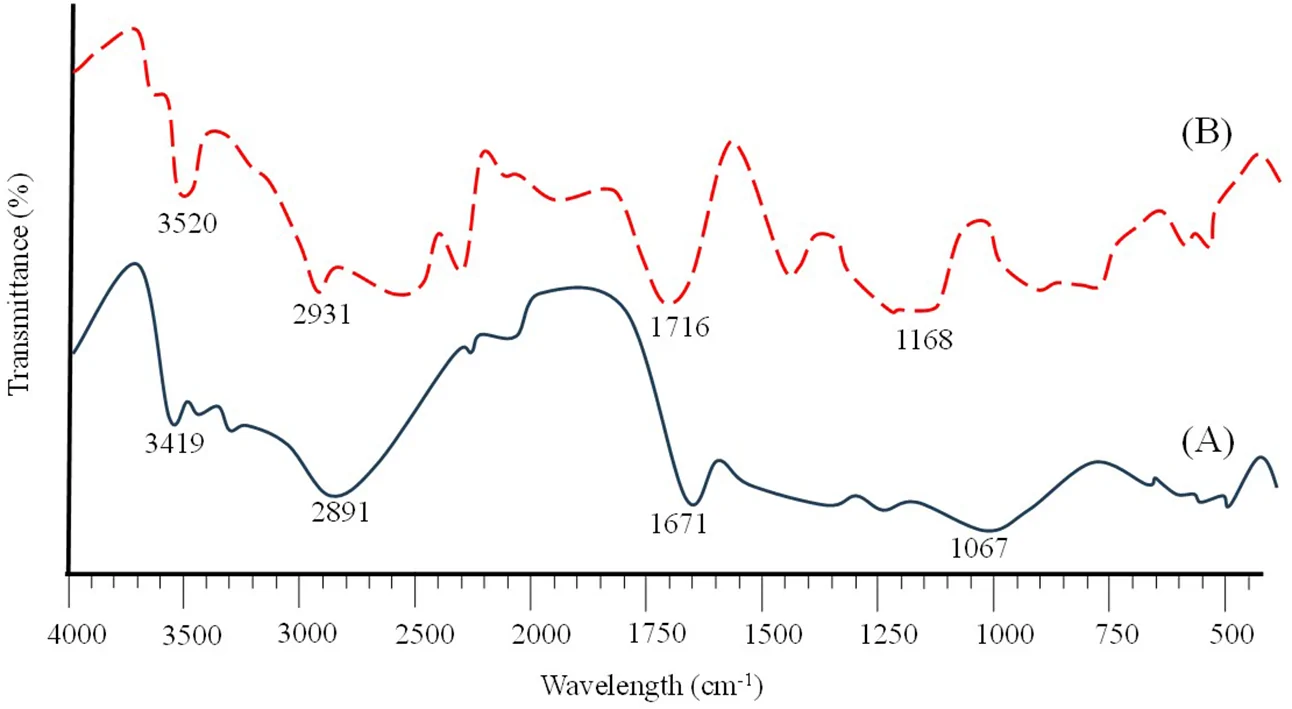
FTIR spectra of CM (A) and CM-co-MA (B) showing the successful synthesis of the CM-co-MA.

CM-co-MA was successfully synthesized by treating CM (polymer) with MA (monomer) in the presence of APS (initiator) and MBA (crosslinker) using the free radical copolymerization method. The FTIR spectrum of CM indicated the presence of a prominent peak at 3419 cm^-1^, due to the presence of hydroxyl (-OH) functionality, at 2891 cm^-1^ due to the presence of -CH and -CH_2_ signals of CM, at 1671 cm^-1^ because of the presence of carbonyl (-C = O) functional group of carboxylic acid (-COOH) revealed the presence of uronic acid moieties in CM, and at 1067 cm^-1^ due to the presence of the C-O-C functional group confirming the glycosidic linkage in the polymeric chains of CM (Fig 1A) [30]. The successful synthesis of CM-co-MA by free radical polymerization of CM with MA was shown by the development of a new signal at 1716 cm^-1^ in the FTIR spectrum of CM-co-MA (Fig 1B), which is due to the formation of ester linkage [44].

### Hemocompatibility studies

The hemolytic index of CM-co-MA was found to be 4.07%. According to the safety standards of ISO document 10993–4:2017, the value of the hemolytic index should be less than 5% if the material is intended to be used for biomedical applications and considered a biocompatible material. However, this value is close to the upper limit, and caution should be exercised in future applications. Slight variations in material preparation or batch differences could influence hemolytic potential. Therefore, although CM-co-MA meets the current safety standards, ongoing monitoring and optimization are recommended for clinical translation. The thrombogenic potential of CM-co-MA was assessed by measuring the weight of the blood clots formed by the sample and control. The thrombose concentration (%) was found to be 86.28 ± 1.98%, indicating that CM-co-MA is non-thrombogenic. Based on these studies, CM-co-MA is considered a safe material for various biomedical applications. The high thrombus reduction value (86.28 ± 1.98%) indicates reduced clot formation relative to the positive control, demonstrating favorable blood compatibility and low thrombogenic potential of CM-co-MA. Materials with lower thrombus adhesion and formation are generally considered more suitable for biomedical and wound-healing applications involving blood contact.

### Antioxidant activities

#### DPPH assay.

**Fig 2:**
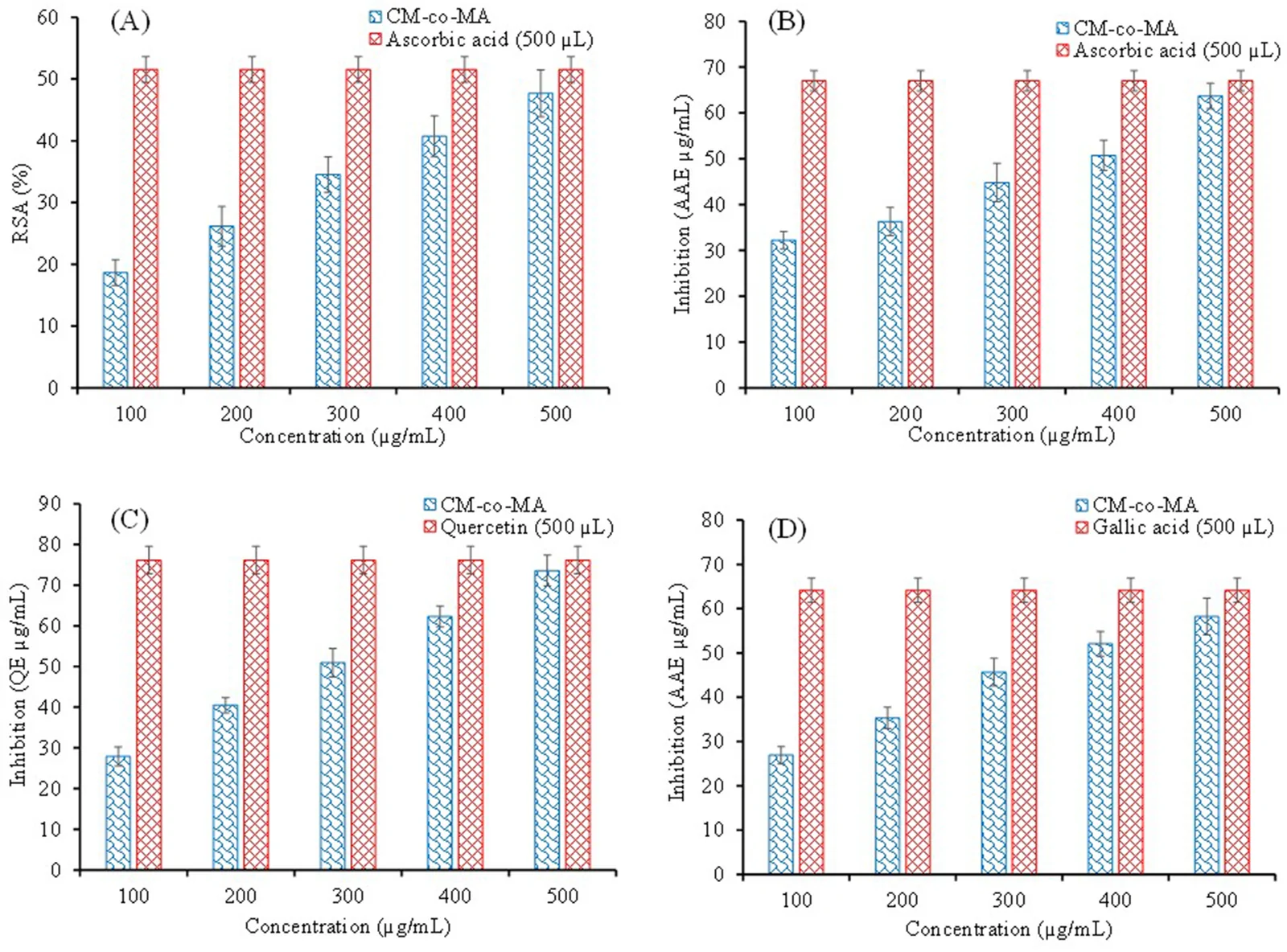
Results of the antioxidant activities of the CM-co-MA: DPPH (A), FRAP (B), TPC (C), and TFC (D). Data are presented as mean ± standard deviation (SD) from three independent replicates (n = 3).

The DPPH assay is a widely used and reliable method for evaluating the antioxidant capacity of chemical compounds. Therefore, a DPPH test was conducted to examine the potential of CM-co-MA to scavenge DPPH free radicals at concentrations ranging from 100–500 µg/mL. Based on their ability to neutralize DPPH radicals, their antioxidant efficacy was compared with that of ascorbic acid, a conventional antioxidant. As the concentration of CM-co-MA increased from 100 to 500 µg/mL, the DPPH radical inhibition percentage also increased from 18.68 to 47.70%. These results imply that CM-co-MA could affect the radical-scavenging mechanism (Fig 2A). As the concentration of CM-co-MA increases, more antioxidant active sites (such as -OH and -COOH groups) become available, leading to greater donation of electrons or hydrogen atoms and resulting in higher DPPH radical inhibition. This dose-dependent behavior has been consistently observed across various antioxidant studies, where radical scavenging activity increases with concentration [45,46].

#### FRAP assay.

The antioxidant activity of CM-co-MA was measured using an ascorbic acid standard curve and based on its reducing ability. The radical scavenging activity ranged from 32.22 to 63.70 µg (expressed as AAE µg/mL) across concentrations of 100–500 µg/mL (Fig 2B). The antioxidant potential of CM-co-MA is based on the stabilization of free radicals, including direct proton transfer, sequential proton loss followed by electron transfer, and hydrogen atom transfer. Ascorbic acid was selected as the reference antioxidant because of its ability to neutralize free radicals and inhibit the propagation of oxidative chain reactions. Its potency arises from the high density of free hydroxyl and polyhydroxyl groups, which readily scavenge reactive species. Trichloroacetic acid solution was used to eliminate concentrated potassium ferrocyanide (K_3_Fe(CN)_6_). When FeCl_3_ was added, a green-to-blue complex was formed, indicating the reduction of Fe^3+^ to Fe^2+^.

#### TPC and TFC assay.

Across the tested concentrations, TPC values increased from 26.95 to 58.21 µg GAE/mL (Fig 2C), and TFC values changed from 27.91 to 73.59 µg QE/mL (Fig 2D). At the higher concentration of CM-co-MA, the results were comparable to those of the respective standards, *i.e*., gallic acid for TPC and quercetin for TFC, indicating that the phenolic and flavonoid content of CM-co-MA was of similar magnitude to that of well-recognized antioxidant compounds. This similarity in values suggests that CM-co-MA retains a rich profile of polyphenolic and flavonoid compounds capable of donating electrons or hydrogen atoms to neutralize free radicals, thereby interrupting oxidative chain reactions. In practical terms, this means that CM-co-MA could deliver antioxidant performance approaching that of pure reference antioxidants. Such activity is directly relevant to biomedical and pharmaceutical applications, where antioxidant-rich materials are known to protect tissues from oxidative damage, reduce inflammation, and promote healing. Moreover, the presence of high TPC and TFC supports CM-co-MA in expanding its potential for future use in drug delivery systems, wound dressings, nutraceutical formulations, and cosmeceutical products. These applications could leverage both the structural properties of CM-co-MA as a hydrogel copolymer and its inherent antioxidant profile to provide dual benefits, such as mechanical protection and biochemical defense against oxidative stress. The antioxidant activity of CM-co-MA was compared with standard antioxidant compounds for reference purposes only. However, because CM-only and uncrosslinked control formulations were not evaluated in the present study, the precise contribution of individual hydrogel components to the observed antioxidant activity requires further investigation.

### Antibacterial activities

**Fig 3:**
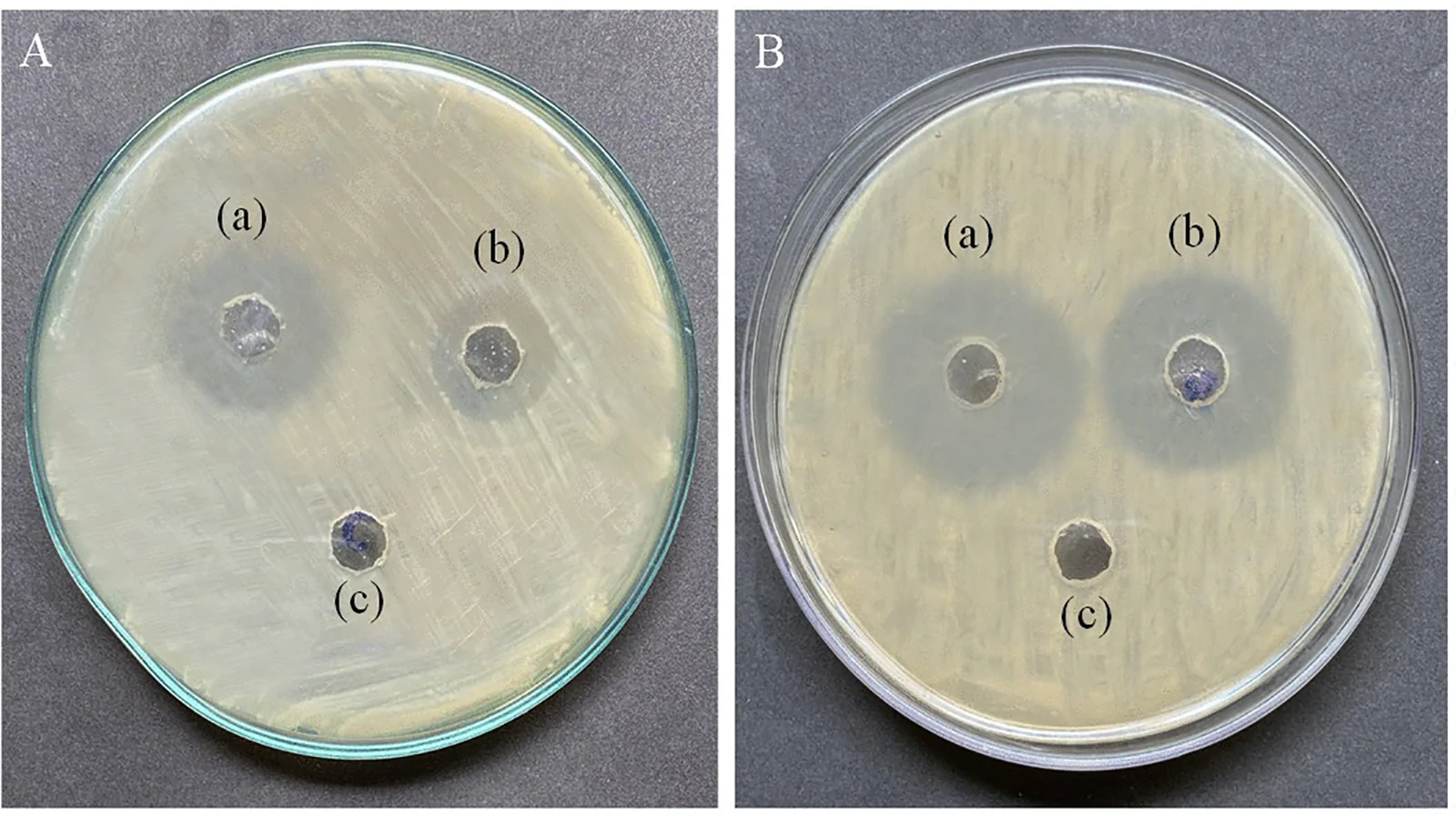
Antibacterial activities of CM-co-MA against Gram-negative (*E. coli*) (A) and Gram-positive (*L. monocytogenes*) (B) bacterial strains, where positive control (a), CM-co-MA (sample) (b), and negative control (c).

**Fig 4:**
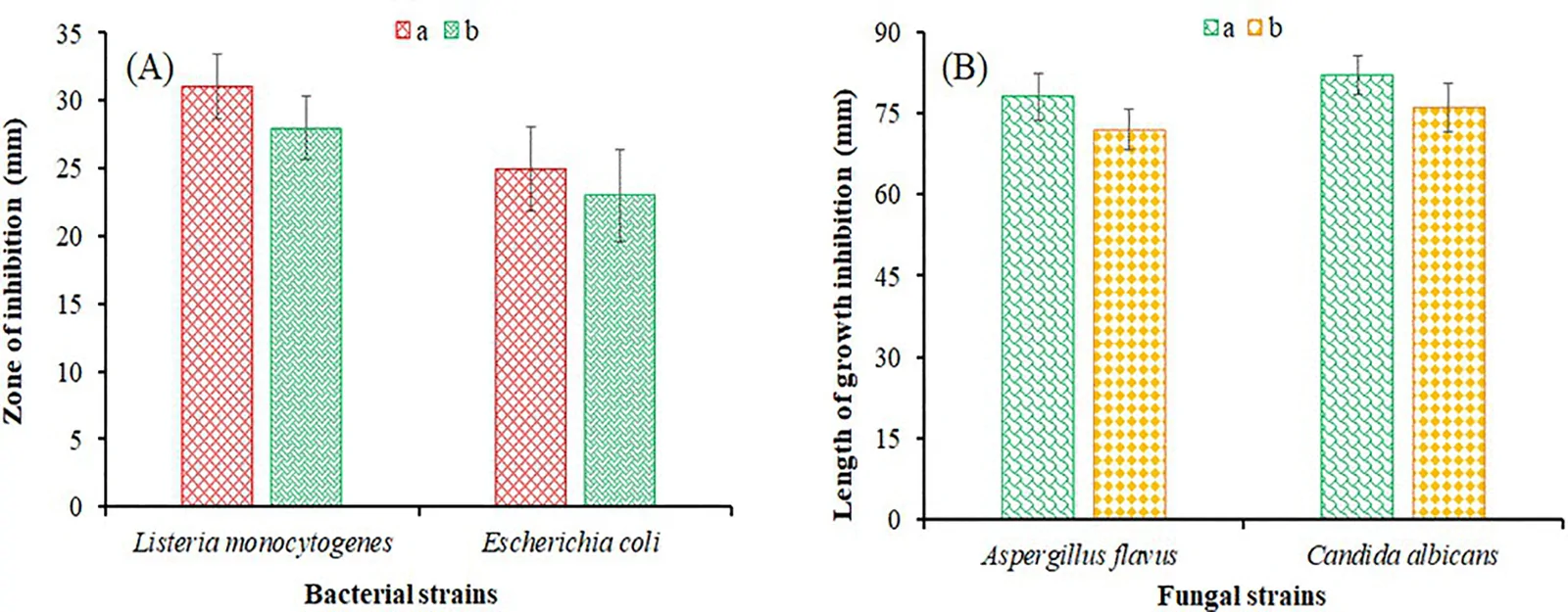
Antimicrobial activities of CM-co-MA against bacterial (A) and fungal (B) strains showing length of growth inhibition along the tube of positive control (a) and CM-co-MA (b). Values are expressed as mean ± SD of three replicates (n = 3). Statistical significance was determined by one-way ANOVA followed by Tukey’s HSD test (p < 0.05).

The rapid emergence of antibiotic-resistant bacteria has become a critical global health concern, diminishing the effectiveness of conventional antibiotics [47]. The significant antibacterial activity of CM-co-MA offers a promising alternative strategy to combat resistant strains. Unlike traditional antibiotics, which often target specific pathways and allow pathogens to develop resistance over time, polysaccharide-based copolymers exert their effects through multiple mechanisms, including physical disruption of cell membranes, interference with nutrient transport, and inhibition of essential enzymatic functions. Such multifaceted modes of action make the development of resistance less likely to occur. Moreover, CM-co-MA can be employed in combination with existing antibiotics to achieve synergistic effects, potentially reducing the required antibiotic dose and slowing resistance development. These properties position CM-co-MA as a potential component in future antimicrobial formulations aimed at addressing the pressing challenge of antibiotic resistance. The antibacterial activity of CM-co-MA was evaluated using the well diffusion method against both gram-negative (*E. coli*) (Fig 3A) and gram-positive (*L. monocytogenes*) (Fig 3B) bacterial strains. The copolymer (CM-co-MA) exhibited notable bactericidal potential, producing a mean zone of inhibition (ZOI) of 27.87 ± 0.2^b^ mm for *L. monocytogenes* and 23.00 ± 0.23^b^ mm for *E. coli*. In comparison, the positive control (Ciprofloxacin) produced ZOIs of 31.00 ± 0.22 ^a^ mm and 25.00 ± 0.23^a^ mm, respectively. Statistical analysis via Tukey HSD test confirmed that the results were highly significant (p=0.0001 for *L. monocytogenes* and p = 0.0005 for *E. coli*). Statistical analysis via Tukey HSD test confirmed that the bactericidal potential of CM-co-MA was highly significant compared to the control group, as indicated by the different superscript letters (^a, b^) (Fig 3A, B, and Fig 4A). The higher susceptibility of *L. monocytogenes* to CM-co-MA may be attributed to the simpler cell wall architecture of gram-positive bacteria, which lack the outer lipopolysaccharide membrane found in gram-negative species such as *E. coli*. This structural difference allows CM-co-MA to penetrate more effectively and interact with the thick peptidoglycan layer, resulting in greater disruption of cellular function. Conversely, the outer membrane of *E. coli* acts as an additional permeability barrier, diminishing the overall antibacterial response of CM-co-MA. These findings indicate that CM-co-MA can either kill or suppress the growth of bacterial pathogens and could serve as a promising candidate for developing alternative antimicrobial agents. Similar observations on the antimicrobial potential of polysaccharide-based copolymers have been reported [39].

### Antifungal activities

The antifungal potential of CM-co-MA was evaluated against two pathogenic fungi: *A. flavus* and *C. albicans*. Two treatments were assessed: standard voriconazole (positive control, labeled “a”) and sample CM-co-MA (labeled “b”). The maximum length of growth inhibition was measured in mm, with mean values and statistical data presented in Fig 4B. For *A. flavus*, voriconazole produced the largest growth inhibition (78 mm), followed closely by CM-co-MA (72 mm). A similar trend was observed for *C. albicans*, where the crude extract, Voriconazole, and CM-co-MA exhibited growth inhibition of 82 mm and 76 mm, respectively. These findings demonstrate that CM-co-MA possesses potent antifungal activity, approaching the efficacy of standard antifungal drugs. The enhanced bioactivity is due to the polysaccharide nature of the extract, which facilitates interactions with fungal cells. These observed effects may result from multiple mechanisms, including reactive oxygen species (ROS) generation, disruption of cell membrane integrity, and inhibition of key enzymatic pathways, ultimately compromising fungal growth and viability. Given their strong activity against *A. flavus*, a major aflatoxin producer, and *C. albicans*, a clinically significant yeast, these CM-co-MAs hold promise as broad-spectrum antifungal agents, particularly in the context of increasing resistance to conventional antifungal therapies.

### Acute oral toxicity tests

The safety of active and inactive pharmaceutical ingredients, materials/chemicals used for coating biomedical devices and surgical equipment, and materials used for implants must be evaluated to authenticate the non-toxic, non-irritant, non-immunogenic, and non-mutagenic nature of these materials. One such parameter to determine the non-toxic and non-irritant nature of biomedical materials is through acute toxicity testing. Therefore, CM-co-MA was evaluated for acute oral toxicity, acute dermal toxicity, and eye irritation.

#### Physical, behavioral, and psychological observations.

None of the animals in the experimental groups exhibited any abnormal behavioral changes, *i.e.,* as biting, leaping, drowsiness, confusion, aggression, self-harm, self-isolation, or extreme hoarding, during the 14-day acute oral toxicity study. All rabbits were healthy and active following oral delivery of CM-co-MA. Impaired mobility, vomiting, seizures, diarrhea, increased salivation, allergic symptoms, skin rashes, and pigmentation were analyzed, and the rabbits in the experimental groups (CM2, CM3, and CM4) lacked any hypersensitivity indications.

### Determination of body weight

One of the most significant and sensitive markers for toxicity determination is a continuous decrease in body weight. The weights of the rabbits slightly decreased over the first three days, but this was quickly reversed. Less food was consumed during the first three days after oral CM-co-MA administration, which may have contributed to weight reduction. The rabbits began to gain weight over the week, which is a sign of normal animal development and physiological function. Furthermore, it was determined that the difference between the control and experimental group animals was statistically insignificant during the whole course of the study (Table 1).

**Table 1:** Bodyweight (g) of rabbits of the control and experimental groups (mean ± SD).

Parameters	Group “CM1”	Group “CM2”	Group “CM3”	Group “CM4”
Pretreatment	1212.52 ± 21.71	1269.67 ± 23.09	1251.15 ± 27.04	1287.77 ± 28.73
Day 1	1214.17 ± 17.35	1265.21 ± 23.53	1248.51 ± 21.55	1283.51 ± 23.70
Day 2	1218.77 ± 23.19	1262.22 ± 28.35	1246.04 ± 26.87	1280.38 ± 29.51
Day 3	1223.41 ± 20.16	1268.32 ± 26.59	1241.64 ± 29.23	1277.71 ± 27.82
Day 7	1234.22 ± 26.62	1282.44 ± 31.44	1246.31 ± 29.19	1297.47 ± 27.25
Day 14	1244.61 ± 21.14	1297.41 ± 26.63	1255.25 ± 21.13	1311.20 ± 29.31

*p < 0.05 indicates a significant difference compared to the control.

### Consumption of water

The water consumption of the control and experimental groups did not differ significantly (Table 2). Rabbits in Groups CM3 and CM4 consumed slightly less water on day 1, which may be due to the ingestion of a large amount of CM-co-MA. After days 7 and 14, water consumption returned to normal.

**Table 2:** Mean values of water consumption (mL) of the control and experimental groups of rabbits.

Parameters	Group “CM1”	Group “CM2”	Group “CM3”	Group “CM4”
Pretreatment	20.18 ± 1.23	20.34 ± 1.25	22.24 ± 1.61	24.67 ± 1.32
Day 1	21.98 ± 1.12	19.91 ± 1.17	21.33 ± 1.51	20.98 ± 1.41
Day 2	22.33 ± 1.79	19.69 ± 2.28	20.18 ± 1.93	18.72 ± 1.78
Day 3	23.26 ± 1.36	20.34 ± 1.47	21.26 ± 1.98	17.32 ± 1.81
Day 7	26.12 ± 1.70	22.64 ± 1.82	25.15 ± 2.69	22.12 ± 1.88
Day 14	29.13 ± 1.95	25.37 ± 2.23	29.72 ± 1.36	27.19 ± 2.82

*p < 0.05 indicates a significant difference compared to the control.

### Consumption of food

The amount of food consumed by the rabbits in the control and experimental groups is presented in Table 3. A statistically insignificant difference in food consumption was observed between the rabbits in the control and experimental group was observed. Until day 3 (Table 3), rabbits in groups CM3 and CM4 consumed slightly less food, most probably due to the fullness of the stomach after ingestion of CM-co-MA. After days 7 and 14, increased food intake was observed, indicating normal digestive system functioning. These results suggest that the gastrointestinal tract and all relevant organs are functioning properly.

**Table 3:** Mean values of food consumption (g) of control and experimental groups of rabbits (mean ± SD).

Parameters	Group “CM1”	Group “CM2”	Group “CM3”	Group “CM4”
Pretreatment	20.44 ± 1.15	21.65 ± 1.39	21.13 ± 1.11	22.96 ± 2.30
Day 1	21.45 ± 2.08	20.13 ± 1.49	20.46 ± 1.82	19.67 ± 1.38
Day 2	22.09 ± 1.94	20.56 ± 1.64	19.21 ± 1.76	17.28 ± 1.55
Day 3	22.91 ± 2.13	21.23 ± 2.09	20.59 ± 1.67	16.32 ± 1.76
Day 7	24.51 ± 1.41	23.59 ± 1.99	24.36 ± 1.96	22.75 ± 1.85
Day 14	28.28 ± 2.81	25.78 ± 1.29	27.73 ± 1.70	25.23 ± 2.29

### Survival rate

It was noted that, even at the maximum tested dose of 2 g/kg of the body weight of rabbits, none of the rabbits died. This indicates that CM-co-MA is a safe material, even at high doses. According to the Globally Harmonized System of Classification and Labelling of Chemicals (GHS), any material with an LD_50_ value higher than 2 g/kg is categorized as a category 5 substance, *i.e.*, a substance with a low acute toxicity level; therefore, CM-co-MA can be recognized as a less toxic material.

### Hematological analysis

The bone marrow is responsible for the production of blood cells; therefore, any agent that disturbs the functioning of the bone marrow will affect different parameters of CBC. The results of the hematological parameters are presented in Table 4. All parameters of the experimental group animals were comparable to those of the control group animals, and no significant differences were observed between the two groups.

**Table 4:** Hematological parameters of rabbits.

Parameters	Group “CM1”	Group “CM2”	Group “CM3”	Group “CM4”
TLC (× 10^3^ µL^-1^)	3.8	4.4	4.1	4.0
RBC (× 10^6^ µL^-1^)	4.1	4.2	4.5	4.3
Hb (g dL^-1^)	13.8	12.7	13.1	14.1
HCT (PCV) (%)	41.8	43.2	45.9	41.1
MCV (fL)	75.3	78.1	71.2	88.3
MCH (pg)	24.4	29.1	32.3	30.4
MCHC (g dL^-1^)	31.6	29.8	30.9	31.9
Platelet count (× 10^3^ µL^-1^)	182	168	177	180
Neutrophil (%)	60.7	63.9	66.4	67.1
Lymphocyte (%)	30.5	27.9	24.8	23.8
Monocyte (%)	5.4	4.8	5.9	5.2
Eosinophil (%)	3.4	3.4	2.9	3.9

### Biochemical analysis

Serum biochemical studies were performed to assess the possible detrimental effects of CM-co*-*MA on vital organs. Important hepatic biochemical markers, including serum enzymes, *i.e.,* as aspartate aminotransferase (AST), alanine aminotransferase (ALT), alkaline phosphatase (ALP), and total bilirubin, are vital indicators of liver function. Any change in the levels of these biochemical markers indicates hepatotoxicity. Similarly, kidney performance can be evaluated using blood urea and creatinine levels.

CM-co-MA did not cause any significant harm to the liver or kidney function, as indicated by the lack of discernible alterations in enzyme levels, urea, or creatinine. All measured parameters were either within the normal range or similar to those of the control group, confirming the non-toxicity of CM*-*co-MA (Table 5). Moreover, there were no variations in electrolyte levels compared to the control. Any significant fluctuation in serum electrolyte concentrations may lead to major health issues, particularly cardiovascular emergencies. Furthermore, the lipid profile was within permissible limits and comparable to that of the control group animals. These results indicate the safety of oral administration of CM*-*co-MA.

**Table 5:** Biochemical parameters of rabbits.

Parameters	Group “CM1”	Group “CM2”	Group “CM3”	Group “CM4”
*Lipid profile*				
Cholesterol (mg dL^-1^)	122	119	115	129
Triglyceride (mg dL^-1^)	76	83	87	80
HDL (mg dL^-1^)	55	62	47	43
LDL (mg dL^-1^)	71	84	81	89
VLDL (mg dL^-1^)	13	15	16	13
*Liver function test*				
Bilirubin (mg dL^-1^)	0.6	0.8	0.9	0.6
SGPT (ALT) (U L^-1^)	21.3	27.9	33.0	30.5
SGOT (AST) (U L^-1^)	31.4	28.5	22.8	33.9
ALP (U L^-1^)	55.1	63.0	67.2	70.4
Total protein (g dL^-1^)	7.1	7.5	6.9	7.9
Albumin (g dL^-1^)	3.4	4.3	4.1	4.5
Globulin (g dL^-1^)	2.3	2.1	2.9	2.6
*Renal function test*				
Urea (mg dL^-1^)	18.7	21.3	23.9	19.9
Creatinine (mg dL^-1^)	0.8	0.9	1.1	1.1
*Serum electrolyte*				
Potassium (mmol L^-1^)	3.9	4.3	4.1	4.2
Sodium (mmol L^-1^)	133	129	144	138
Uric acid (mg dL^-1^)	4.5	4.1	4.9	5.1

### Absolute organ body weight

The weights of the vital organs of the rabbits in the experimental and control groups were noted and compared. The results suggested that the absolute organ weights of the rabbits in the experimental and control groups did not differ significantly (Table 6).

**Table 6:** Absolute organ weight (g) of rabbits of the control and experiment group (mean ± SD).

Parameters	Group “CM1”	Group “CM2”	Group “CM3”	Group “CM4”
Heart	4.88 ± 0.51	4.52 ± 0.88	5.05 ± 0.84	5.11 ± 0.81
Kidney	18.48 ± 1.33	20.33 ± 0.67	21.11 ± 1.91	22.31 ± 1.76
Stomach	17.81 ± 0.39	18.49 ± 0.98	19.56 ± 1.05	19.97 ± 0.84
Intestine	45.91 ± 3.11	49.47 ± 4.33	52.33 ± 5.15	55.41 ± 5.55
Liver	51.44 ± 4.41	55.28 ± 4.94	58.73 ± 6.19	59.32 ± 7.12

### Histopathology and gross necropsy

**Fig 5:**
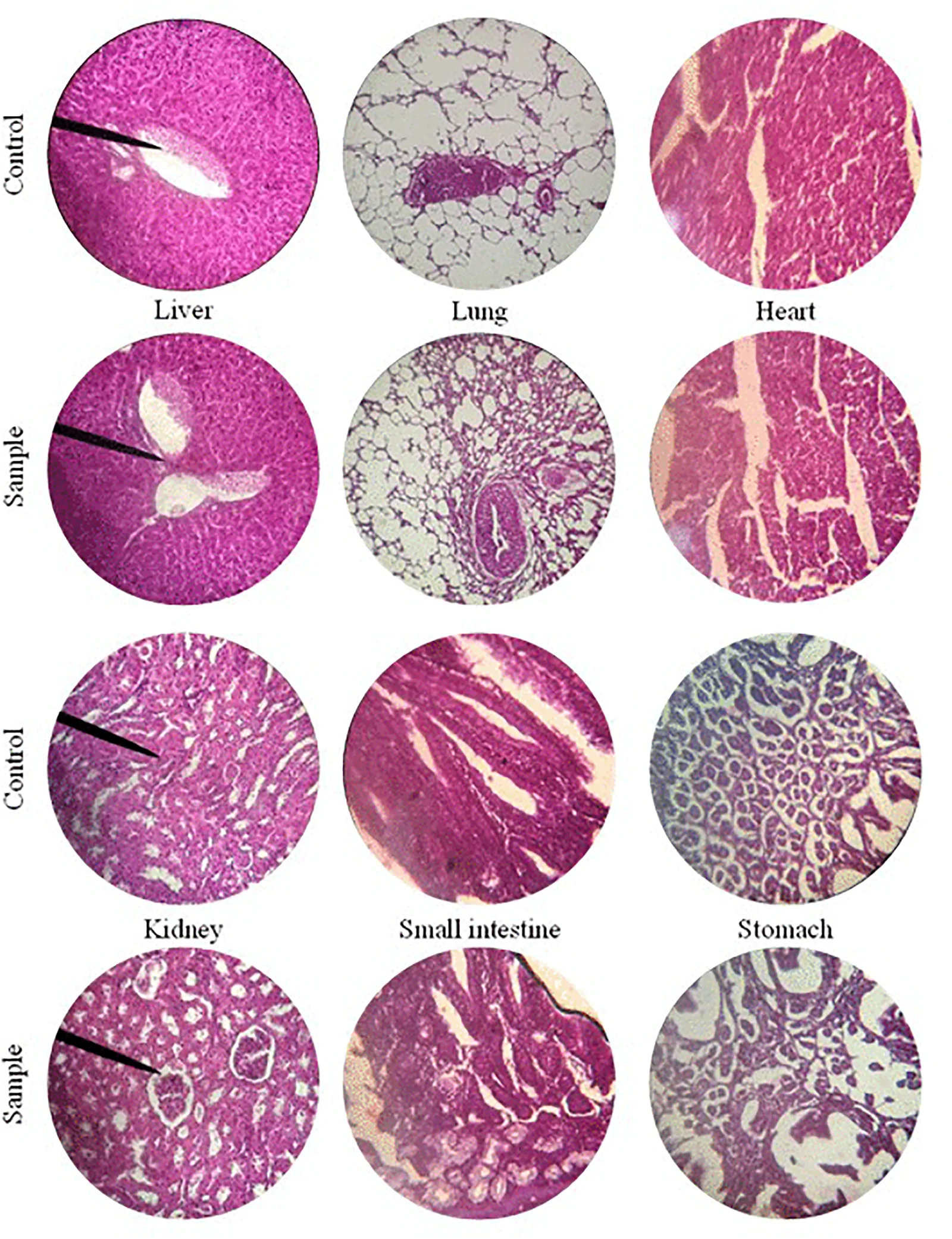
Histopathology of liver, lungs, heart, kidney, small intestine, and stomach of the rabbits of the control and sample (2 g/kg body weight) groups.

The vital organs of the rabbits, *i.e.*, lungs, liver, heart, kidneys, stomach, and small intestine, showed histopathological evidence of unchanged cellular structures following oral treatment with CM*-*co-MA. The histological slides of the vital organs are shown in Fig 5. The cellular/tissue structures of these vital organs displayed the absence of inflammation, degeneration, lesions, and necrosis.

### Dermal toxicity testing

The lack of dermal toxicity of CM*-*co-MA was demonstrated by the absence of rashes, inflammation, lesions, abrasion, allergy, erythema, and infection during the acute dermal toxicity study, which also confirmed the non-irritating and non-toxic nature of the substance.

### Primary eye irritation

After applying CM-co-MA to the eyes of the rabbits, there were no signs of discomfort, irritation, inflammation, redness, lacrimation, or dryness of the eyes. Since none of the examined animals had any form of inflammation, irritation, or conjunctivitis, they were all assigned a score of “0” on the Draize scale. Although the acute oral, dermal, and eye irritation studies conducted in this work demonstrate the short-term safety of CM-co-MA, it is acknowledged that cytotoxicity, genotoxicity, and chronic toxicity assessments were not performed in the current study. These evaluations are important for comprehensive preclinical safety profiles. This limitation has now been explicitly stated, and future studies are planned to address these additional toxicity parameters to ensure a thorough safety assessment for potential biomedical applications.

### Wound-healing capability

**Fig 6:**
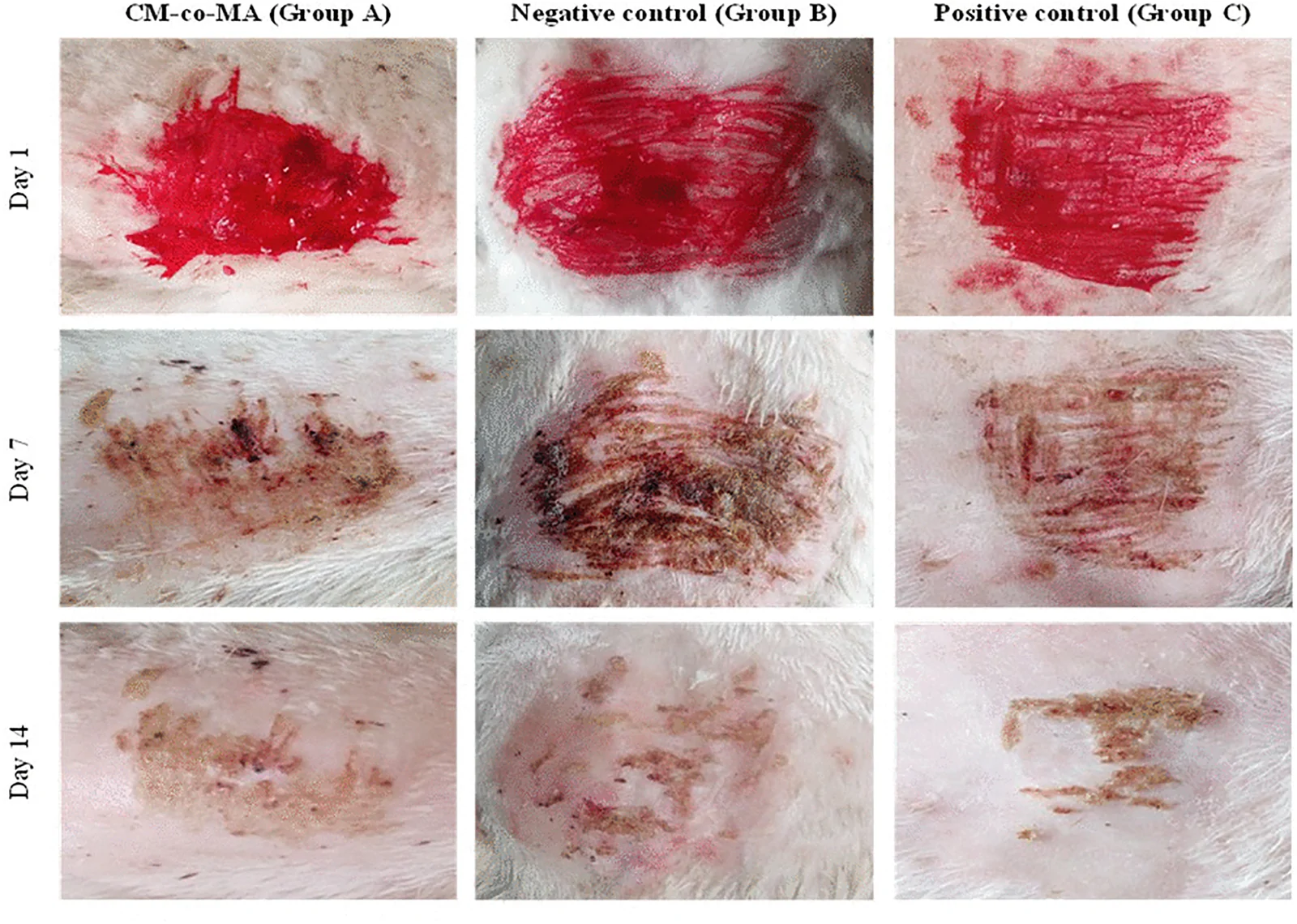
Wound healing progression in rabbits treated with CM-co-MA (Group A), negative control (Group B), and positive control (standard *Sufre tulle®* bandage, Group C) monitored on days 1, 7, and 14.

Excision wounds were created on the left leg of the rabbits using a sharp surgical knife. The animals were randomly divided into three treatment groups (n = 6): Group A, CM-co-MA (sample); Group B, negative control (no active treatment); and Group C, standard *Sufre tulle®* bandage. Wound healing progression was monitored, and the percentage of wound closure was calculated on days 1, 7, and 14 of the study period (Fig 6). In the negative control group, wound closure in rabbits was 0.17, 4.39, and 45.50% on days 1, 7, and 14, respectively. In the CM-co-MA-treated group, the wound closure rate was increased markedly to 1.70, 57.65, and 84.40% at the same time intervals. Similarly, in the *Sufre tulle®* bandage group, wound closure rates were 2.60, 70.60, and 97.30%, respectively.

The wound closure data were statistically analyzed using one-way ANOVA followed by Tukey’s HSD test. Both the CM-co-MA-treated group and the positive control group showed significantly higher wound closure percentages compared to the negative control at days 7 and 14 (p < 0.05). On day 14, CM-co-MA achieved 84.40% wound closure, whereas the positive control showed 97.30% wound closure, indicating superior healing compared to the untreated group (45.50%). The healing performance of CM-co-MA was comparable to that of the standard *Sufre tulle®* bandage. Both CM-co-MA and standard *Sufre tulle®* bandages showed significantly faster wound closure than the negative control. These findings suggest that CM-co-MA has significant wound-healing potential and could serve as a viable alternative to commercial wound-healing bandages.

### Histological assessment of wound healing

**Fig 7:**
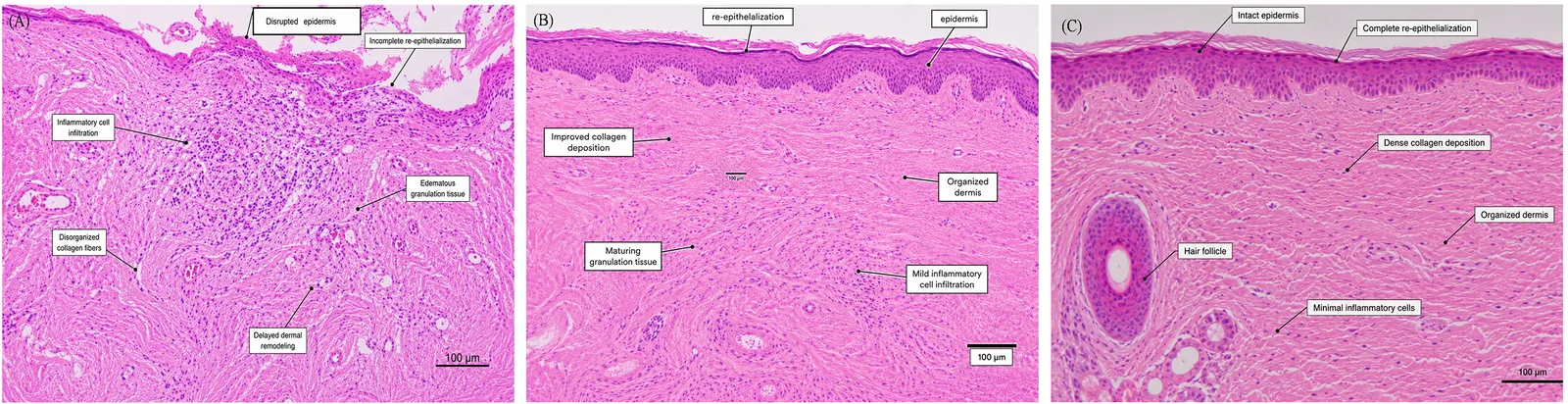
Representative H&E-stained sections showing the wound tissue for (A) negative control, (B) CM-co-MA-treated sample, and (C) positive control (Sufre tulle® bandage).

Histological evaluation of the wound tissue was performed to assess the degree of regeneration and tissue organization across the experimental groups (Fig 7). Fig 7A depicts the negative control, which exhibited a disrupted epidermis, incomplete re-epithelialization, disorganized collagen fibers, delayed dermal remodeling, and pronounced inflammatory cell infiltration, indicating poor and delayed wound repair. Fig 7B shows the CM-co-MA-treated sample, which exhibited re-epithelialization, improved collagen deposition, organized dermis, mild inflammatory cell infiltration, and maturing granulation tissue, demonstrating accelerated tissue repair and structural organization compared to the negative control. Fig 7C, the positive control (Sufre tulle® bandage) displayed an intact epidermis, almost complete re-epithelialization, dense collagen deposition, minimal inflammatory cells, fully organized dermis, and well-formed hair follicles, reflecting optimal wound healing. These histological findings align with the wound closure data, supporting the conclusion that CM-co-MA treatment promotes faster and more structured tissue regeneration, bridging the gap between untreated wounds and standard treatments.

### Mechanism of wound healing

**Scheme 2:**
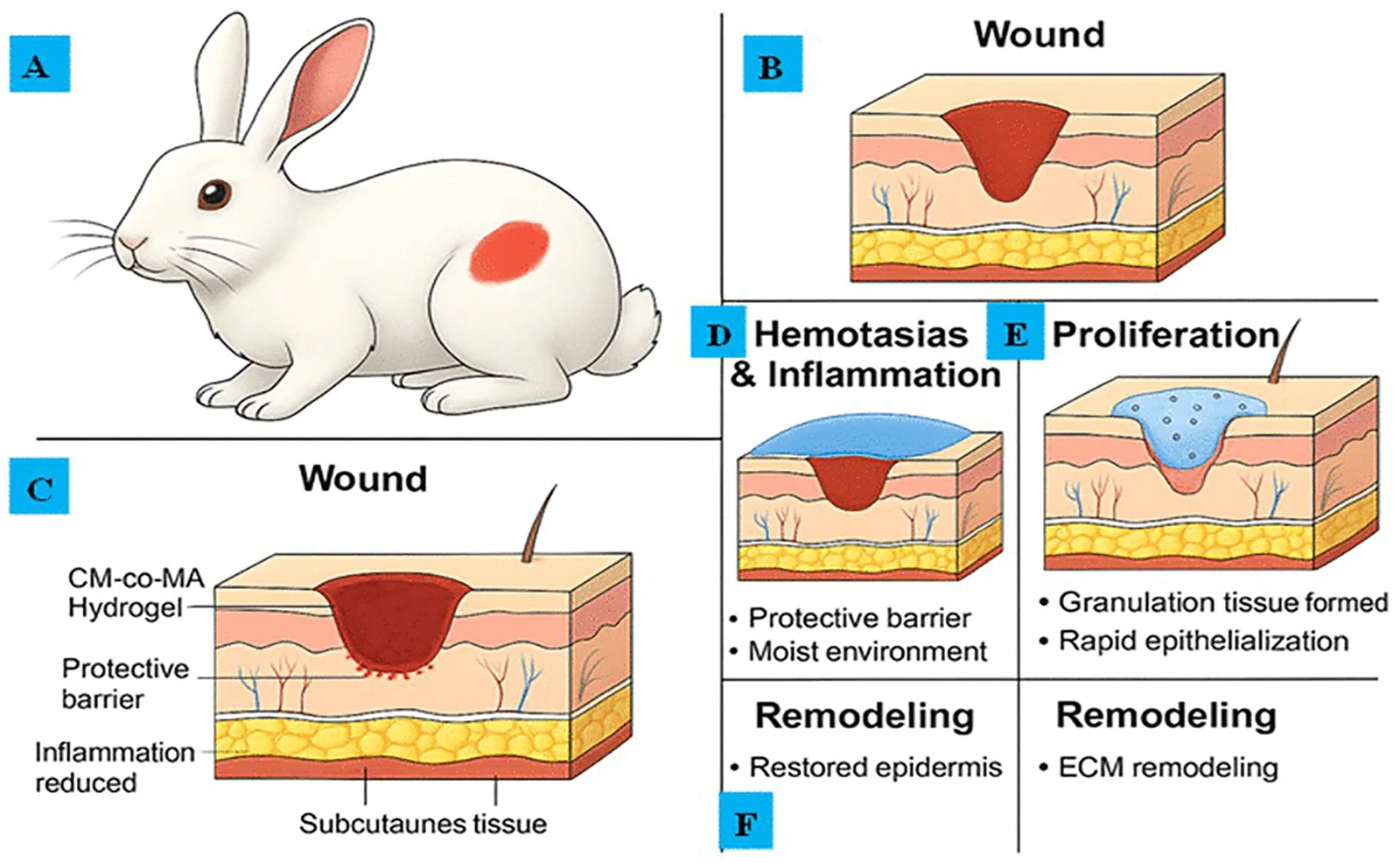
Wound-healing mechanism of CM-co-MA in rabbits. Experimental rabbit model showing wound site (A), cross-sectional view of an untreated wound before hydrogel application (B), application of CM-co-MA forming a protective barrier and reducing inflammation (C), hemostasis and inflammation phase with a moist, protected wound environment (D), proliferation phase showing granulation tissue formation and rapid epithelialization (E), remodeling phase depicting restored epidermis and extracellular matrix (ECM) organization (F).

The wound-healing process with CM-co-MA begins immediately after its application. Upon placement over the wound site, CM-co-MA forms a moist, protective barrier that shields the injured tissue from microbial invasion and mechanical trauma while reducing inflammation. This moist environment prevents dryness, allowing cellular processes to proceed efficiently. The proposed mechanism by which CM-co-MA accelerates wound healing through moisture retention, protection, and stimulation of granulation, epithelialization, and collagen remodeling is shown in Scheme 2.

During the hemostasis and inflammation phases, CM-co-MA maintained wound hydration, supported clot stabilization, and reduced inflammatory responses. This helps prepare the wound bed for the next stage by minimizing tissue damage and promoting an optimal biochemical environment for healing. In the proliferation phase, the CM-co-MA-hydrated, biocompatible matrix facilitates fibroblast proliferation, collagen deposition, and angiogenesis, leading to granulation tissue formation. The moist interface also accelerates keratinocyte migration, enabling rapid epithelial coverage of the wound.

Finally, in the remodeling phase, the new epithelium is strengthened through extracellular matrix (ECM) reorganization and collagen fiber alignment, restoring skin integrity and tensile strength. By supporting all four phases, *i.e.*, from protection and inflammation control to tissue regeneration and maturation, CM-co-MA accelerates wound closure and achieves healing outcomes comparable to those of standard commercial dressings.

## Conclusion

A copolymer hydrogel (CM-co-MA) was successfully synthesized and applied for wound healing. The CM-co-MA showed non-hemolytic and non-thrombogenic features, indicating its biocompatibility. CM-co-MA possesses remarkable anti-oxidant, antibacterial, and anti-fungal activities. CM*-*co*-*MA was tested for toxicity *in vivo* in rabbits, and the results revealed no significant changes in the physical behavior, number of hematological, biochemical, and histological parameters. CM-co-MA was applied to the eyes of rabbits and was found to be a safe material. The wound-healing capability of CM*-*co-MA was exceptionally high when applied to the skin of rabbits. In conclusion, the results of all these studies indicated that the CM-co-MA is a biocompatible, antioxidant, antimicrobial, antifungal, and non-toxic biomaterial with outstanding wound healing efficacy. Therefore, developing a safe drug delivery system for oral and topical administration of antibacterial and antifungal drugs is recommended. Nonetheless, additional toxicological examinations are still needed, including mutagenic testing, cytotoxicity, and chronic toxicity tests.
