# Supplementary material for: Synthesis and evaluation of haemocompatible, antioxidant, and antibacterial properties, and acute oral toxicity of a methacrylic acid mediated glucoxylan for wound healing applications
Source: PLoS One. 2026 Aug 27;21(8):e0356647. doi: 10.1371/journal.pone.0356647 (PMC13521390; doi:10.1371/journal.pone.0356647)
Supplement: Peer review history

# Peer review: Decision Letter 0

**DOI:** 10.1371/journal.pone.0356647.r001

**Authors:** Salem Salem

5 Apr 2026

Dear Dr. Vandy,

Thank you for submitting your manuscript to PLOS ONE. After careful consideration, we feel that it has merit but does not fully meet PLOS ONE’s publication criteria as it currently stands. Therefore, we invite you to submit a revised version of the manuscript that addresses the points raised during the review process.

==============================

**ACADEMIC EDITOR:  Major Revision**

Please submit your revised manuscript by May 17 2026 11:59PM. If you will need more time than this to complete your revisions, please reply to this message or contact the journal office at plosone@plos.org. When you're ready to submit your revision, log on to https://www.editorialmanager.com/pone/ and select the 'Submissions Needing Revision' folder to locate your manuscript file.

- A letter that responds to each point raised by the academic editor and reviewer(s). You should upload this letter as a separate file labeled 'Response to Reviewers'.
- A marked-up copy of your manuscript that highlights changes made to the original version. You should upload this as a separate file labeled 'Revised Manuscript with Track Changes'.
- An unmarked version of your revised paper without tracked changes. You should upload this as a separate file labeled 'Manuscript'.

If you would like to make changes to your financial disclosure, please include your updated statement in your cover letter. Guidelines for resubmitting your figure files are available below the reviewer comments at the end of this letter.

If applicable, we recommend that you deposit your laboratory protocols in protocols.io to enhance the reproducibility of your results. Protocols.io assigns your protocol its own identifier (DOI) so that it can be cited independently in the future. For instructions see: https://journals.plos.org/plosone/s/submission-guidelines#loc-laboratory-protocols. Additionally, PLOS ONE offers an option for publishing peer-reviewed Lab Protocol articles, which describe protocols hosted on protocols.io. Read more information on sharing protocols at https://plos.org/protocols?utm_medium=editorial-email&utm_source=authorletters&utm_campaign=protocols.

As the corresponding author, your ORCID iD is verified in the submission system and will appear in the published article. PLOS supports the use of ORCID, and we encourage all coauthors to register for an ORCID iD and use it as well. Please encourage your coauthors to verify their ORCID iD within the submission system before final acceptance, as unverified ORCID iDs will not appear in the published article. *Only*  the individual author can complete the verification step; PLOS staff *cannot*  verify ORCID iDs on behalf of authors.

We look forward to receiving your revised manuscript.

Kind regards,

Salem S. Salem

Academic Editor

PLOS One

Journal requirements:

When submitting your revision, we need you to address these additional requirements.

1. Please ensure that your manuscript meets PLOS ONE's style requirements, including those for file naming. The PLOS ONE style templates can be found at

https://journals.plos.org/plosone/s/file?id=wjVg/PLOSOne_formatting_sample_main_body.pdf and

https://journals.plos.org/plosone/s/file?id=ba62/PLOSOne_formatting_sample_title_authors_affiliations.pdf

2. Thank you for stating the following in the Acknowledgments Section of your manuscript:

“The authors express their appreciation to the Deanship of Scientific Research at King Khalid University, Saudi Arabia, for this work through a research group program under grant number RGP-2/528/46.”

We note that you have provided funding information that is not currently declared in your Funding Statement. However, funding information should not appear in the Acknowledgments section or other areas of your manuscript. We will only publish funding information present in the Funding Statement section of the online submission form.

Please remove any funding-related text from the manuscript and let us know how you would like to update your Funding Statement. Currently, your Funding Statement reads as follows:

“The author(s) received no specific funding for this work.”

Please include your amended statements within your cover letter; we will change the online submission form on your behalf.

3. Your ethics statement should only appear in the Methods section of your manuscript. If your ethics statement is written in any section besides the Methods, please delete it from any other section.

If the reviewer comments include a recommendation to cite specific previously published works, please review and evaluate these publications to determine whether they are relevant and should be cited. There is no requirement to cite these works unless the editor has indicated otherwise.

[Note: HTML markup is below. Please do not edit.]

Reviewers' comments:

Reviewer's Responses to Questions

**Comments to the Author**

1. Is the manuscript technically sound, and do the data support the conclusions?

Reviewer #1: Yes

Reviewer #2: Partly

**********

2. Has the statistical analysis been performed appropriately and rigorously?

Reviewer #1: No

Reviewer #2: N/A

**********

3. Have the authors made all data underlying the findings in their manuscript fully available?

The PLOS Data policy

Reviewer #1: Yes

Reviewer #2: Yes

**********

4. Is the manuscript presented in an intelligible fashion and written in standard English?

Reviewer #1: Yes

Reviewer #2: No

**********

Reviewer #1: The foundational concept of this study is scientifically sound and the breadth of characterization is commendable. However, the manuscript in its current form contains several concerns that undermine the validity of its core claims:

1. A paired t-test compares only two related measurements; with multiple experimental groups, one-way ANOVA followed by post-hoc testing (e.g., Tukey's HSD or Bonferroni correction) is required.

2. The antioxidant data (Fig. 2) are presented without any statistical comparison between CM-co-MA and the reference standards at each concentration point.

3. The wound healing data (Fig. 6) contain no statistical analysis whatsoever. Wound closure percentages (e.g., 84.40% vs. 97.30% on Day 14) are presented as raw numbers with no test of significance.

4. Fungal ZOI values exceeding 70 mm are extraordinarily large and demand explanation or correction

5. Only one concentration (40 µg/mL) was tested for antibacterial activity, while antioxidant assays used 5 concentrations

6. No power calculation or statistical justification for sample sizes is provided (n=6 for toxicity, n=3 for wound healing)

7. n = 3 per group is critically underpowered for an in vivo wound healing study. This sample size cannot support meaningful statistical inference or account for biological variability between animals

8. The abstract claims ~95% recovery in treated rabbits after 14 days. However, the Results section (Section 3.9) reports the CM-co-MA group achieved only 84.40% closure at Day 14, while the Sufre tulle® standard achieved 97.30%

9. Histological analysis of wound tissue is entirely absent. The authors include organ histopathology for toxicity assessment but provide no histological evaluation of the wound site itself

10. The thrombose concentration is reported as 86.28 ± 1.98%, explain the reference ranges used and why this value supports non-thrombogenicity.

11. The hemolytic index of 4.07% is close to the ISO 10993-4:2017 threshold of 5%. The authors should discuss this proximity to the safety limit more critically

12. The title uses "glucoxylan" while the abstract and body consistently use "glucomannan." These are not synonymous

13. Figure 2 axes are mislabeled — the x-axis states "Concentration (µL)" when the units should be µg/mL as described in the methods.

14. Figures 2A and 2B use identical y-axis labels ("Inhibition (AAE µg/mL)") despite measuring different parameters (DPPH inhibition % vs. FRAP reducing capacity).

15. The dose for anesthesia during blood collection is not specified separately from the euthanasia dose (60 mg/kg i.p.). Anesthetic and euthanasia doses are typically distinct.

Reviewer #2: “Synthesis and evaluation of cicatrizant properties, biomedical applications, and acute oral toxicity of a methacrylic acid mediated glucoxylan for wound healing applications”

This manuscript presents the synthesis of a chia seed mucilage/methacrylic acid copolymeric hydrogel and its evaluation for wound healing applications. The topic is timely and relevant, and the authors provide an extensive dataset, particularly regarding acute toxicity and in vivo safety. However, the study suffers from unclear novelty, limited material characterization relative to the claims made, and overinterpretation of biological results. Several methodological aspects, especially in antimicrobial testing and wound healing evaluation, require stronger rigor.

A major revision is necessary before the work can be considered for publication. The authors may find the following comments helpful for revision of their paper.

- Novelty is not clearly defined. Similar CM-based hydrogels, including MA-grafted systems, have already been reported. The manuscript should clarify whether the contribution lies in material design or extended biological validation and adjust claims accordingly.

- Characterization is insufficient for a wound dressing material. FTIR alone does not support claims related to moisture retention, mechanical performance, or barrier function. Key properties such as swelling behavior, mechanical strength, and stability are missing.

- Direct comparison of hydrogel antioxidant activity with pure standards (gallic acid, quercetin, ascorbic acid) is misleading. The source of antioxidant activity is unclear, and the absence of CM only controls weakens interpretation.

- Antifungal inhibition zones are unusually large and raise concerns about methodology and interpretation. Reliance on zone of inhibition data alone is insufficient, and claims of strong or drug comparable activity should be moderated.

- In vivo wound healing study is underpowered (n = 3 per group). Statistical analysis is limited, and no histological assessment of healed tissue is provided. Conclusions regarding tissue regeneration are preliminary.

- Acute oral, dermal, and eye irritation studies are a strength, but they only address short-term safety. The manuscript should more clearly acknowledge the absence of cytotoxicity, genotoxicity, and chronic toxicity data, if possible.

- The manuscript requires substantial language editing, improved figure clarity, consistent terminology, and a more concise discussion.

**********

what does this mean?). If published, this will include your full peer review and any attached files.

If you choose “no”, your identity will remain anonymous but your review may still be made public.

**Do you want your identity to be public for this peer review?**  For information about this choice, including consent withdrawal, please see our Privacy Policy

Reviewer #1: No

Reviewer #2: **Yes:**  Mohamed F. Attia

**********

[NOTE: If reviewer comments were submitted as an attachment file, they will be attached to this email and accessible via the submission site. Please log into your account, locate the manuscript record, and check for the action link "View Attachments". If this link does not appear, there are no attachment files.]

To ensure your figures meet our technical requirements, please review our figure guidelines: https://journals.plos.org/plosone/s/figures

You may also use PLOS’s free figure tool, NAAS, to help you prepare publication quality figures: https://journals.plos.org/plosone/s/figures#loc-tools-for-figure-preparation.

NAAS will assess whether your figures meet our technical requirements by comparing each figure against our figure specifications.

# Author comment: Author response to Decision Letter 1

**DOI:** 10.1371/journal.pone.0356647.r002

2 Jun 2026

The authors would like to sincerely thank the Editor and the Reviewers for their careful evaluation of our manuscript and for their valuable comments and constructive suggestions. Their insightful feedback has significantly improved the quality, clarity, and scientific rigor of the manuscript. All comments have been carefully considered, and the manuscript has been revised accordingly. We hope that the revisions satisfactorily address all concerns raised by the reviewers and editor.

Journal requirements:

When submitting your revision, we need you to address these additional requirements.

1. Please ensure that your manuscript meets PLOS ONE's style requirements, including those for file naming. The PLOS ONE style templates can be found at

https://journals.plos.org/plosone/s/file?id=wjVg/PLOSOne_formatting_sample_main_body.pdf and

https://journals.plos.org/plosone/s/file?id=ba62/PLOSOne_formatting_sample_title_authors_affiliations.pdf

2. Thank you for stating the following in the Acknowledgments Section of your manuscript:

“The authors express their appreciation to the Deanship of Scientific Research at King Khalid University, Saudi Arabia, for this work through a research group program under grant number RGP-2/528/46.”

We note that you have provided funding information that is not currently declared in your Funding Statement. However, funding information should not appear in the Acknowledgments section or other areas of your manuscript. We will only publish funding information present in the Funding Statement section of the online submission form.

Please remove any funding-related text from the manuscript and let us know how you would like to update your Funding Statement. Currently, your Funding Statement reads as follows:

“The author(s) received no specific funding for this work.”

Please include your amended statements within your cover letter; we will change the online submission form on your behalf.

3. Your ethics statement should only appear in the Methods section of your manuscript. If your ethics statement is written in any section besides the Methods, please delete it from any other section.

If the reviewer comments include a recommendation to cite specific previously published works, please review and evaluate these publications to determine whether they are relevant and should be cited. There is no requirement to cite these works unless the editor has indicated otherwise.

5. Review Comments to the Author

Please use the space provided to explain your answers to the questions above. You may also include additional comments for the author, including concerns about dual publication, research ethics, or publication ethics. (Please upload your review as an attachment if it exceeds 20,000 characters)

Reviewer #1: The foundational concept of this study is scientifically sound and the breadth of characterization is commendable. However, the manuscript in its current form contains several concerns that undermine the validity of its core claims:

1. A paired t-test compares only two related measurements; with multiple experimental groups, one-way ANOVA followed by post-hoc testing (e.g., Tukey's HSD or Bonferroni correction) is required.

Authors response: We thank the reviewer for this valuable comment. The statistical analysis section has been revised accordingly. The previously mentioned paired t-test approach has been replaced with one-way analysis of variance (ANOVA) followed by Tukey’s Honestly Significant Difference (HSD) post-hoc test for multiple group comparisons. The revised statistical methodology is now clearly described in the “Statistical analysis” section of the manuscript

2. The antioxidant data (Fig. 2) are presented without any statistical comparison between CM-co-MA and the reference standards at each concentration point.

Authors response: We thank the reviewer for this valuable comment. The statistical analysis section has been revised accordingly.

3. The wound healing data (Fig. 6) contain no statistical analysis whatsoever. Wound closure percentages (e.g., 84.40% vs. 97.30% on Day 14) are presented as raw numbers with no test of significance.

Authors response: Thank you dear reviewer updated and revised.

4. Fungal ZOI values exceeding 70 mm are extraordinarily large and demand explanation or correction

Authors response: We thank the reviewer for highlighting this point. The antifungal activity of CM-co-MA was evaluated using the macro-dilution method, in which fungal growth occurs in a liquid medium within test tubes. In this method, the length of growth inhibition along the tube is measured rather than a diffusion zone on agar. This approach allows complete inhibition along the medium and can produce larger numerical values compared to standard agar-based assays. All experiments were conducted in triplicate, and reported values represent the mean inhibition length. We have clarified this methodology in the revised Methods section (2.8) and Discussion (3.5) to avoid misinterpretation.

5. Only one concentration (40 µg/mL) was tested for antibacterial activity, while antioxidant assays used 5 concentrations

Authors response: We appreciate the reviewer’s observation. The antibacterial activity of CM-co-MA was evaluated at a single concentration (40 µg/mL) as a preliminary assessment to confirm its potential against Gram-positive and Gram-negative bacteria. This approach is commonly used for initial screening in polysaccharide-based copolymers, allowing for clear observation of zones of inhibition without extensive optimization at this stage. In contrast, antioxidant assays require multiple concentrations (100–500 µg/mL) to establish dose-dependent activity and enable quantitative comparison with standard antioxidants. We have clarified this rationale in the Methods section (2.7) and the Discussion (3.4) of the revised manuscript. Dose-response studies for antibacterial activity may be considered in future investigations.

6. No power calculation or statistical justification for sample sizes is provided (n=6 for toxicity, n=3 for wound healing)

Authors response: We thank the reviewer for this valuable comment. The in vivo wound-healing study was conducted using six animals per treatment group (n = 6), as stated in the revised manuscript. Similarly, the acute toxicity studies were also performed with n = 6 animals per group. The selected sample sizes were based on commonly reported preliminary in vivo biomedical studies and ethical considerations aimed at minimizing animal use while maintaining statistical reliability.

7. n = 3 per group is critically underpowered for an in vivo wound healing study. This sample size cannot support meaningful statistical inference or account for biological variability between animals

Authors response: We thank the reviewer for this comment. We would like to clarify that this was a typographical error in the manuscript. Each treatment group actually contained 6 rabbits (n = 6), while there were 3 groups in total (Negative Control, CM-co-MA, and Standard Bandage). The Methods section and figure legends have been revised to accurately reflect n = 6 per group,

8. The abstract claims ~95% recovery in treated rabbits after 14 days. However, the Results section (Section 3.9) reports the CM-co-MA group achieved only 84.40% closure at Day 14, while the Sufre tulle® standard achieved 97.30%

Authors response: We thank the reviewer for pointing out this inconsistency. The value reported in the abstract (~95%) was a typographical error. The correct wound closure percentage for the CM-co-MA-treated group at Day 14 is 84.40 ± 3.0%, while the standard Sufre tulle® bandage group reached 97.30 ± 3.0%, consistent with the Results section (3.9) and revised Figure 6. The abstract has now been updated to accurately report 84% closure for CM-co-MA-treated rabbits, ensuring consistency with the main text and statistical analyses.

9. Histological analysis of wound tissue is entirely absent. The authors include organ histopathology for toxicity assessment but provide no histological evaluation of the wound site itself

Authors response: We thank the reviewer for this important comment. We have now included histological evaluation of the wound tissue at Day 14 for all experimental groups. Representative H&E-stained sections have been added as Figure 7.

10. The thrombose concentration is reported as 86.28 ± 1.98%, explain the reference ranges used and why this value supports non-thrombogenicity.

Authors response: We thank the reviewer for this important comment. The thrombogenicity result (86.28 ± 1.98%) reflects the thrombus reduction percentage relative to the positive control, where higher values indicate lower thrombus formation and improved blood compatibility. Materials exhibiting reduced thrombus formation are considered less thrombogenic and more suitable for biomedical applications involving blood contact. To clarify this interpretation, additional explanatory text has now been added to the revised manuscript, together with a clearer description of the reference control and the significance of the obtained value.

11. The hemolytic index of 4.07% is close to the ISO 10993-4:2017 threshold of 5%. The authors should discuss this proximity to the safety limit more critically

Authors response: We thank the reviewer for this observation. The hemolytic index of 4.07% for CM-co-MA is below the ISO 10993-4:2017 threshold, indicating the material is non-hemolytic and biocompatible. However, we acknowledge that this value is close to the upper safety limit. Minor variations in material preparation or batch differences could influence hemolytic potential. This critical discussion has been added to the revised manuscript (Section 3.2) to emphasize both compliance with standards and caution for future applications.

12. The title uses "glucoxylan" while the abstract and body consistently use "glucomannan." These are not synonymous

Authors response: We thank the reviewer for highlighting this inconsistency. The term “glucomannan” used in the abstract and body was incorrect and resulted from an inadvertent typographical error. The correct polysaccharide investigated throughout the study is “glucoxylan,” and not “glucomannan.” To maintain consistency and scientific accuracy, the terminology has been carefully checked throughout the manuscript to ensure uniform usage of “glucoxylan” in all sections.

13. Figure 2 axes are mislabeled — the x-axis states "Concentration (µL)" when the units should be µg/mL as described in the methods.

Authors response: We thank the reviewer for pointing out this labeling error. The x-axis of Figure 2 has been revised and updated to correctly reflect the units as µg/mL, consistent with the concentrations described in the Methods section (2.6). The figure now accurately represents the data for all antioxidant assays.

14. Figures 2A and 2B use identical y-axis labels ("Inhibition (AAE µg/mL)") despite measuring different parameters (DPPH inhibition % vs. FRAP reducing capacity).

Authors response: We thank the reviewer for noting this oversight. The y-axis labels of Figures 2A and 2B have been revised and updated to accurately reflect the measured parameters.

15. The dose for anesthesia during blood collection is not specified separately from the euthanasia dose (60 mg/kg i.p.). Anesthetic and euthanasia doses are typically distinct.

Authors response: We thank the reviewer for this important observation. We clarify that rabbits were anesthetized for blood collection using sodium pentobarbital at 30 mg/kg i.p., which provides sufficient surgical anesthesia without causing mortality. Euthanasia was performed at a higher dose of 60 mg/kg i.p. to ensure humane sacrifice. Both doses were applied following institutional ethical guidelines and ARRIVE standards. The Methods section (2.4 and 2.9) has been updated in the revised manuscript to clearly distinguish the anesthetic and euthanasia doses.

Reviewer #2

Reviewer #2: “Synthesis and evaluation of cicatrizant properties, biomedical applications, and acute oral toxicity of a methacrylic acid mediated glucoxylan for wound healing applications”

This manuscript presents the synthesis of a chia seed mucilage/methacrylic acid copolymeric hydrogel and its evaluation for wound healing applications. The topic is timely and relevant, and the authors provide an extensive dataset, particularly regarding acute toxicity and in vivo safety. However, the study suffers from unclear novelty, limited material characterization relative to the claims made, and overinterpretation of biological results. Several methodological aspects, especially in antimicrobial testing and wound healing evaluation, require stronger rigor.

A major revision is necessary before the work can be considered for publication. The authors may find the following comments helpful for revision of their paper.

- Novelty is not clearly defined. Similar CM-based hydrogels, including MA-grafted systems, have already been reported. The manuscript should clarify whether the contribution lies in material design or extended biological validation and adjust claims accordingly.

Authors response: The study’s novelty lies in the combination of CM-co-MA hydrogel synthesis with comprehensive biological validation, including hemocompatibility, antioxidant, antimicrobial, toxicity, and in vivo wound healing studies. While similar hydrogels have been reported, this integrated preclinical evaluation has not been previously described. The Introduction have been updated to reflect this accurately.

- Characterization is insufficient for a wound dressing material. FTIR alone does not support claims related to moisture retention, mechanical performance, or barrier function. Key properties such as swelling behavior, mechanical strength, and stability are missing.

Authors response: We sincerely thank the reviewer for this valuable comment. We agree that the comprehensive characterization, including swelling behaviour, mechanical properties, moisture retention, and barrier performance, is important for evaluating wound dressing materials. However, we respectfully clarify that the primary objective of the present study is not to provide an exhaustive structural elucidation, but rather to demonstrate the functional performance and wound healing behaviour of the developed system. In the current manuscript, FTIR analysis is used as a supportive and rapid verification tool to confirm the presence of key functional groups and chemical interactions (e.g., ester linkage formation), which are sufficient for the scope of this study. However, the detailed structural characterization of this material, we already have reported in our previous publication [https://doi.org/10.1016/j.ijbiomac.2026.151609]. To avoid unnecessary repetition and duplication of previously published data, these analyses were not reproduced in the current manuscript.

- Direct comparison of hydrogel antioxidant activity with pure standards (gallic acid, quercetin, ascorbic acid) is misleading. The source of antioxidant activity is unclear, and the absence of CM only controls weakens interpretation.

Authors response: We thank the reviewer for this insightful comment. The antioxidant standards (ascorbic acid, gallic acid, and quercetin) were included primarily as reference compounds to provide comparative benchmarking of the antioxidant potential of CM-co-MA rather than to imply equivalent mechanisms or potency. We agree that the absence of CM-only and uncrosslinked control groups limits precise identification of the specific source of antioxidant activity within the hydrogel system. This limitation has now been acknowledged in the revised manuscript. Future studies will include appropriate material controls to better distinguish the individual contributions of CM, MA, and the copolymer network toward the observed antioxidant activity.

- Antifungal inhibition zones are unusually large and raise concerns about methodology and interpretation. Reliance on zone of inhibition data alone is insufficient, and claims of strong or drug comparable activity should be moderated.

Authors response: We thank the reviewer for highlighting this point. The antifungal activity of CM-co-MA was evaluated using the

Attachment Submitted filename: PONE point by point response R1 5-29.docx

# Peer review: Decision Letter 1

**DOI:** 10.1371/journal.pone.0356647.r003

**Authors:** Salem Salem

6 Aug 2026

Synthesis and evaluation of haemocompatible, antioxidant, and antibacterial properties, and acute oral toxicity of a methacrylic acid mediated glucoxylan for wound healing applications

PONE-D-25-68420R1

Dear Dr. Vandy,

We’re pleased to inform you that your manuscript has been judged scientifically suitable for publication and will be formally accepted for publication once it meets all outstanding technical requirements.

Within one week, you’ll receive an e-mail detailing the required amendments. When these have been addressed, you’ll receive a formal acceptance letter and your manuscript will be scheduled for publication.

An invoice will be generated when your article is formally accepted. Please note, if your institution has a publishing partnership with PLOS and your article meets the relevant criteria, all or part of your publication costs will be covered. Please make sure your user information is up-to-date by logging into Editorial Manager at Editorial Manager® and clicking the ‘Update My Information' link at the top of the page. For questions related to billing, please contact billing support.

If your institution or institutions have a press office, please notify them about your upcoming paper to help maximize its impact. If they’ll be preparing press materials, please inform our press team as soon as possible -- no later than 48 hours after receiving the formal acceptance. Your manuscript will remain under strict press embargo until 2 pm Eastern Time on the date of publication. For more information, please contact onepress@plos.org.

Kind regards,

Salem S. Salem

Academic Editor

PLOS One

Additional Editor Comments (optional):

Reviewers' comments:

Reviewer's Responses to Questions

**Comments to the Author**

Reviewer #1: All comments have been addressed

Reviewer #2: All comments have been addressed

**********

2. Is the manuscript technically sound, and do the data support the conclusions?

Reviewer #1: Yes

Reviewer #2: Partly

**********

3. Has the statistical analysis been performed appropriately and rigorously?

Reviewer #1: Yes

Reviewer #2: N/A

**********

4. Have the authors made all data underlying the findings in their manuscript fully available?

The PLOS Data policy

Reviewer #1: Yes

Reviewer #2: Yes

**********

5. Is the manuscript presented in an intelligible fashion and written in standard English?

Reviewer #1: Yes

Reviewer #2: Yes

**********

Reviewer #1: (No Response)

Reviewer #2: After careful review, I see the authors addressed my questions adequately, therefore, I recommend publication in PLOS ONE in its present form.

**********

what does this mean?). If published, this will include your full peer review and any attached files.

If you choose “no”, your identity will remain anonymous but your review may still be made public.

**Do you want your identity to be public for this peer review?**  For information about this choice, including consent withdrawal, please see our Privacy Policy

Reviewer #1: No

Reviewer #2: No

**********

# Editor report: Acceptance letter

**DOI:** 10.1371/journal.pone.0356647.r004

**Authors:** Salem Salem

PONE-D-25-68420R1

PLOS One

Dear Dr. Vandy,

I'm pleased to inform you that your manuscript has been deemed suitable for publication in PLOS One. Congratulations! Your manuscript is now being handed over to our production team.

At this stage, our production department will prepare your paper for publication. This includes ensuring the following:

* All references, tables, and figures are properly cited

* All relevant supporting information is included in the manuscript submission,

* There are no issues that prevent the paper from being properly typeset

You will receive further instructions from the production team, including instructions on how to review your proof when it is ready. Please keep in mind that we are working through a large volume of accepted articles, so please give us a few days to review your paper and let you know the next and final steps.

Lastly, if your institution or institutions have a press office, please let them know about your upcoming paper now to help maximize its impact. If they'll be preparing press materials, please inform our press team within the next 48 hours. Your manuscript will remain under strict press embargo until 2 pm Eastern Time on the date of publication. For more information, please contact onepress@plos.org.

You will receive an invoice from PLOS for your publication fee after your manuscript has reached the completed accept phase. If you receive an email requesting payment before acceptance or for any other service, this may be a phishing scheme. Learn how to identify phishing emails and protect your accounts at https://explore.plos.org/phishing.

If we can help with anything else, please email us at customercare@plos.org.

Thank you for submitting your work to PLOS One and supporting open access.

Kind regards,

PLOS ONE Editorial Office Staff

on behalf of

Dr. Salem S. Salem

Academic Editor

PLOS One
